# Lignin-Based Catalysts for C–C Bond-Forming Reactions

**DOI:** 10.3390/molecules28083513

**Published:** 2023-04-16

**Authors:** Cristina del Mar García Martín, José Ignacio Hernández García, Sebastián Bonardd, David Díaz Díaz

**Affiliations:** 1 Instituto de Bio-Orgánica Antonio González, Universidad de La Laguna, Avda. Astrofísico Francisco Sánchez 2, 38206 La Laguna, Spain; alu0101066882@ull.edu.es (C.d.M.G.M.); alu0101033525@ull.edu.es (J.I.H.G.); 2 Departamento de Química Orgánica, Universidad de La Laguna, Avda. Astrofísico Francisco Sánchez 3, 38206 La Laguna, Spain

**Keywords:** lignin, catalysis, organic synthesis, C–C bond formation, metal nanoparticles

## Abstract

Carbon–carbon (C–C) bond formation is the key reaction in organic synthesis to construct the carbon framework of organic molecules. The continuous shift of science and technology toward eco-friendly and sustainable resources and processes has stimulated the development of catalytic processes for C–C bond formation based on the use of renewable resources. In this context, and among other biopolymer-based materials, lignin has attracted scientific attention in the field of catalysis during the last decade, either through its acid form or as a support for metal ions and metal nanoparticles that drive the catalytic activity. Its heterogeneous nature, as well as its facile preparation and low cost, provide competitive advantages over other homogeneous catalysts. In this review, we have summarized a variety of C–C formation reactions, such as condensations, Michael additions of indoles, and Pd-mediated cross-coupling reactions that were successfully carried out in the presence of lignin-based catalysts. These examples also involve the successful recovery and reuse of the catalyst after the reaction.

## 1. Introduction

Carbon–carbon (C–C) bond formation lies at the heart of chemical sciences and constitutes the basis of the biogenesis of nature’s essential molecules [1]. Thus, C–C bond-forming reactions enable the synthesis of a plethora of organic compounds that have a wide range of applications, such as fine chemicals, medicinal and pharmaceutical agents, and agrochemicals [2]. However, science and technology applications continuously shift toward eco-friendly and sustainable resources and processes. Indeed, the general public is currently aware of the hazardous substances used and generated by numerous chemical processes, which has contributed to the foundation of the concept of green and sustainable chemistry [3]. For this reason, the scientific community must be committed to reducing the environmental impact of harmful chemicals while developing milder reaction processes [4].

In this context, catalysis has played a vital role in chemical synthesis at both laboratory and industry scales. The use of catalytic systems enables the reduction ofreaction temperatures and enhances reaction selectivities, avoiding undesired side products. Therefore, catalyst design is critical for the development of greener and more sustainable industrial manufacturing where the recovery and reuse of the catalysts are essential. Thus, heterogeneous catalysis by means of supported catalysts presents a series of important advantages over homogeneous catalysis.

In order to develop non-toxic, recyclable, selective, and economic catalysts, scientists are paying increasing attention to biopolymer-supported metal catalysts, such as cellulose [5], alginate [6], chitosan [7], and starch [8]. It is important to emphasize that the catalytic activity of supported metals depends on the particle size distribution and support–metal interactions [9], which largely depend on the physicochemical nature of the supporting material. To understand the increasing interest in biopolymers in this context, it is necessary to realize that production demands in our modern society have promoted the uncontrolled use of fossil-based resources, contributing to worldwide pollution and climate change. Therefore, the use of widely available biopolymers constitutes a sustainable and eco-friendly alternative to petrochemical resources.

**Figure 1:**
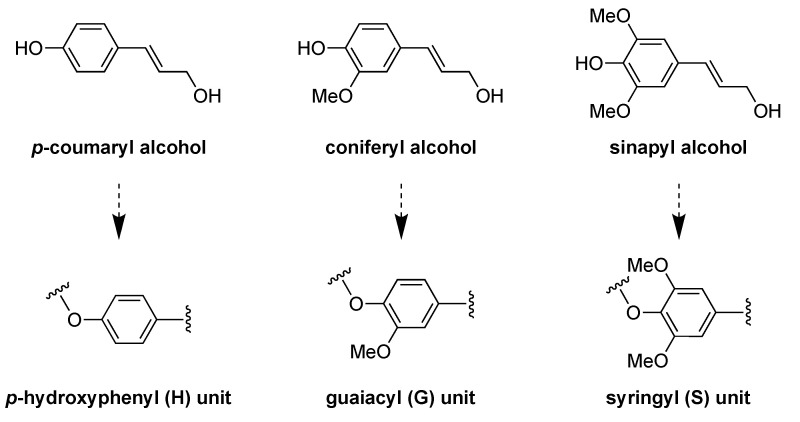
Main three lignin-building blocks (monolignols) (**top**). The corresponding units found in the lignin structure: *p*-hydroxyphenyl (H), guaiacyl (G), and syringyl (S) units (**bottom**).

Among numerous natural polymers used in the field of catalysis, lignocellulose constitutes the second most abundant biomass on Earth and consists of cellulose, hemicellulose, and lignin [10,11,12,13]. While cellulose and hemicellulose have been widely used for numerous applications, lignin has attracted major attention during the last decade [14,15,16,17]. Lignin is a highly aromatic, amorphous, and three-dimensional heteropolymer-building plant cell. It is composed mainly of aliphatic (R−OH) and phenolic (Ph−OH) hydroxyl groups in its backbone, as well as carboxylic and sulfonic groups [18,19]. The main production of lignin derives from the pulp and paper industry, where a huge amount of produced black liquor (ca. 50−70 million tons/year) is burned to supply energy and pulping reagents. Moreover, the molecular structure of lignin is highly dependent on the source and the extraction procedure. In general, it is formed by the radical polymerization of three principal phenylpropanoid monomers (so-called monolignols): coniferyl, sinapyl, and p-coumaryl alcohol. These monomers are synthesized in the cytoplasm and transported to the cell wall, being incorporated in the form of guaiacyl (G), syringyl (S), and *p*-hydroxyphenyl (H) units into the structure of [20,21,22] (Figure 1).

The main extraction method of lignin is the Kraft process for the production of chemical pulp [23,24]. This procedure involves the use of an aqueous solution containing NaOH and sodium sulfide (Na_2_S) to break down lignin–carbohydrate linkages. After 2 h at 170 °C under such alkaline conditions, a solid pulp and a brown liquid (i.e., black liquor) are obtained. Then, cellulose is isolated by purification of the pulp, whereas lignin is precipitated from the black liquor upon acidification. On the other hand, there are also other procedures involving acidic pretreatments that provide water-soluble lignin due to sulfonation. In addition, organosolv treatments are based on hydrothermal treatments with a mixture of water, an organic solvent, and occasionally additional additives to yield high-quality sulfur-free lignin.

In this review, we focus specifically on the preparation and use of lignin-based catalysts for C–C bond-forming reactions. The first section is dedicated to the preparation and main characterization of several lignin catalysts, and the second section is exclusively centered on their use to catalyze a number of chemical reactions leading to C–C bond formation. This review aims to be complementary to previous reviews on the topic where catalysis with lignin-based materials is discussed generally [25,26,27]. In addition, in this review we have incorporated additional C–C bond-forming reactions that are not included in previous contributions.

## 2. Types of Lignin-Based Catalysts

### 2.1. Catalysts Based on Pristine Lignosulfonic Acid (LSA)

**Figure 2:**
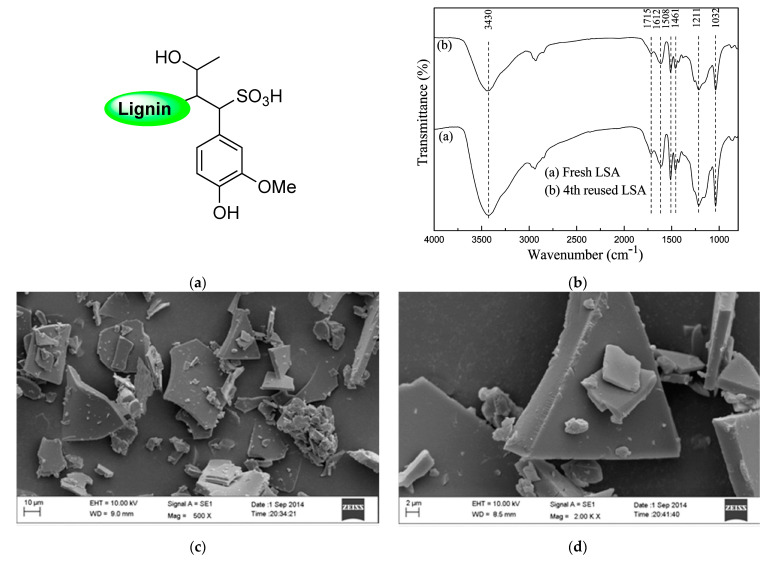
(**a**) Proposed chemical structure of **LSA**. (**b**) FTIR spectra of **LSA** before and after its catalytic evaluation. (**c**,**d**) SEM images of **LSA** at different magnifications. Adapted with permission from reference [31]. Copyright 2015 American Chemical Society.

Due to the enormous number of reports, there is no surprise in finding relevant percentages of sulfur during the elemental analysis of lignin samples. In this sense, it has been well reported that this S content would be ascribed to the presence of sulfonic acid groups covalently attached to the lignin backbone, and one of the most widely accepted explanations for this experimental fact comes from the sulfite pulping processes of Kraft lignin and/or the black liquor acidification step during the recovery of this biopolymer, where sulfuric acid is usually employed [28,29,30]. This type of lignin-bearing sulfonic acid functionality was identified as lignosulfonic acid (**LSA**) or, in its deprotonated form, lignosulfonate, commonly acquired as its sodium salt (**LSA-Na^+^**) (Figure 2a). Even when **LSA** has been largely produced unintentionally, the scientific community has rapidly noted the exceptional properties that this material could provide, namely acting as support for catalytic species or even participating as a catalyst itself. The above is supported by its inherent acid–base property and how this property manages to control several catalytic transformations. As we mentioned before, **LSA** corresponds to the nondeprotonated lignin structure; however, sometimes, the most accessible and commercially available form is deprotonated **LSA-Na^+^**. Therefore, work with **LSA** is commonly preceded by an additional step in which all sulfonate species are returned to their acidic form. Indeed, even in cases where it is possible to acquire **LSA** samples directly, it is recommended to ensure a proper protonation process since the presence of a small portion of sulfonate entities cannot be discarded. An excellent example of the above was performed by Chen and coworkers [31], where prior to assessing the catalytic property of **LSA** samples, they first ensured its protonation by passing an aqueous solution of **LSA-Na^+^** through an activated acidic resin. Then, the obtained **LSA** samples were recovered by freeze-drying. Typically, the characterization of **LSA** specimens is based on infrared spectroscopy (FTIR), elemental analysis (EA), and acid–base titration, techniques that allow the identification and quantification of sulfonic acid functionalities. Regarding FTIR analysis, in addition to the already expected signals accusing OH, COOH, aliphatic, and aromatic portions in lignin, the presence of sulfonate and sulfonic acid groups can be corroborated by signals centered at approximately 1032 cm^−1^ and 1211 cm^−1^, ascribed to the stretching motions of S = O linkages in SO_3_^−^ and SO_3_H entities, respectively, which were also observed after the reutilization of the catalyst [32] (Figure 2b). Of course, after the acidification step, a major contribution of the latest vibrational band is expected. On the other hand, the quantification of sulfonic acids is usually achieved by titration and contrasted with the sulfur content from elemental analysis. Both experiments are expected to give values within the same range; however, slightly lower amounts of SO_3_H were observed in titration experiments. This could be explained by the fact that EA quantifies the total sulfur content without discriminating between SO_3_^−^ and SO_3_H species. Regarding the above, when quantification is needed, both experiments must be carried out in a complementary way. In this regard, Chen and coworkers [31] calculated the SO_3_H density values of 1.97 and 1.88 mmol/g for their **LSA** samples from EA and titration experiments, respectively. In addition, they carried out the morphological characterization of the obtained **LSA** by means of scanning electron microscopy (SEM) analysis (Figure 2c,d), allowing the visualization of granular flakes that were also observed after several catalytic trials.

### 2.2. Catalysts Based on LSA as a Support Matrix for the Immobilization of Transition Metal Ions (LSA-M^n+^)

**Figure 3:**
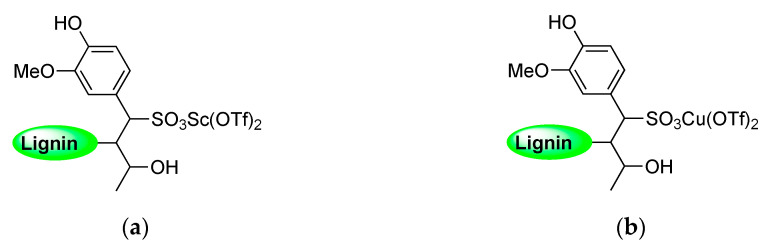
Proposed chemical structures for (**a**) **LSA-Sc^3+^** and (**b**) **LSA-Cu^2+^** catalysts. Adapted with permission from reference [33]. Copyright 2014 Atlanta Publishing House LLC.

The adequate acid–base properties shown by **LSA** allowed its entrance to the catalysis field in a fruitful manner. From this point, the need to expand their catalytic performance toward other chemical transformations became notably attractive in offering a new class of biobased catalysts produced from this highly abundant industrial waste. However, depending only on acid–base properties is not enough to reach catalytic success in different chemical reactions, and from this situation, the inclusion of well-known catalytic transition metals emerged as a suitable strategy to expand the utility of these catalysts by searching for synergistic effects between the **LSA** and catalytic metal ions. In addition, another advantage of this strategy is the reutilization of the material in consecutive reaction cycles due to its insolubility in the reaction media, avoiding the loss of these usually expensive transition metals but also preventing their leakage into the environment. Regarding the above, the first good example of this strategy was achieved in 2013 by Sun and collaborators [33], where they successfully achieved the immobilization of two different metal triflate derivates, Sc(OTf)_3_ and Cu(OTf)_2_, on **LSA-Na^+^** (Figure 3). The authors took advantage of the charged character of **LSA-Na^+^** and separately by a simple ion exchange process which achieved the coordination of both triflate species. Typically, the impregnation process was carried out by dispersing a certain amount of **LSA-Na^+^** into an ethanolic solution of the metal precursor, after which isolation of the hybrid catalyst was easily achieved by filtration, followed by several washing steps, and ending with a final drying stage. It must be mentioned that, in this case, the authors performed the impregnation method at 80 °C and 36 h; however, these parameters should be carefully selected depending on the nature of the metal precursor. Additionally, the authors corroborated the importance of deprotonated sulfonic groups present in the support by observing a direct relation between the metal load and the amount of SO_3_^−^ species. After obtaining the catalyst, the most important characterization corresponds to the quantification of the metal loading by means of inductively coupled plasma mass spectrometry (ICP-MS). In this work, the authors reached loading values of 0.022 and 0.044 mmol/g for **LSA-Sc^3+^** and 0.80 mmol/g for **LSA-Cu^2+,^** which were employed with good results in the catalyzed formation of C–C bonds.

**Figure 4:**
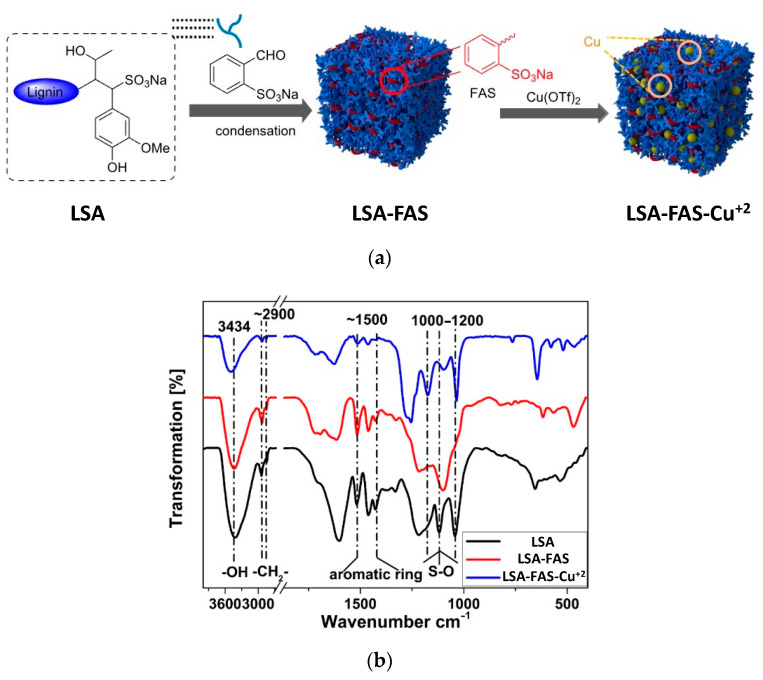
(**a**) Scheme for the preparation of **LSA-FAS** and **LSA-FAS-Cu^2+^** catalysts. (**b**) FTIR spectra of **LSA**, **LSA-FAS**, and **LSA-FAS-Cu^2+^**. Adapted with permission from reference [31]. Copyright 2014 Atlanta Publishing House LLC.

Interestingly, the authors extended this impregnation protocol and successfully tested the immobilization of catalytic nonmetallic species, that is, an amine-functionalized imidazole-based ionic liquid (**LSA-IL@NH_2_**). Although there are no metal ions involved in this case, it constitutes an isolated example described in the same publication [33]. Therefore, we have decided to mention it here rather than creating a separate section. Briefly, the catalyst was achieved after performing the ionic exchange process between ammonium lignosulfonate (**LSA-NH_4_^+^**) and a suitable ionic liquid (IL) derivative [33], allowing the preparation of a catalytic system with an IL loading of 0.48 mmol/g (calculated from EA). Even when the above work stood out by the wide variety of reactions tested, a very limited catalyst characterization was performed, being mostly based on the quantification of metal loadings. Notwithstanding the above, seven years later (2020), some members of the same group involved in the above work contributed a new and improved methodology for the impregnation of Cu^2+^ on **LSA**, along with its catalytic assessment [34]. Additionally, in this opportunity, a more in-depth characterization of the catalyst was performed. The new strategy relied on the increment of sulfonic groups by chemically modifying **LSA** with 2-formylbenzenesulfonic acid sodium (**FAS**) (Figure 4a). To achieve this, the authors carried out condensation reactions between the phenol residues of **LSA** and the aldehyde group of **FAS** entities. The process was performed using water as the solvent and concentrated HCl as the catalyst while stirring at 90 °C and 8 h of reaction, after which the obtained solid (**LSA-FAS**) was isolated by filtration, abundantly washed, and finally dried at 110 °C. The increase of sulfonic groups was constated by EA, from which an increment of approximately 4% was measured for S% regarding unmodified **LSA**. The authors devised the increment of SO_3_H entities as an efficient way to introduce more metal-coordinating sites enhancing the metal fixation on the material. In this regard, they achieved the impregnation of Cu(OTf)_2_ species by dispersing **LSA-FAS** in refluxing ethanol containing the copper precursor. After purifying the **LSA-FAS-Cu^2+^** catalyst, ICP analysis revealed a Cu^2+^ loading of 0.92 mmol/g, which was considerably higher than the values of 0.45 and 0.56 mmol/g reached with two other reference materials, thereby confirming the success of the strategy [34]. The chemical structure of the material was first analyzed by FTIR spectroscopy, allowing the identification of typical signals ascribed to the lignin backbone, especially those accusing the presence of SO_3_^−^ and SO_3_H structures, [35,36] (Figure 4b).

**Figure 5:**
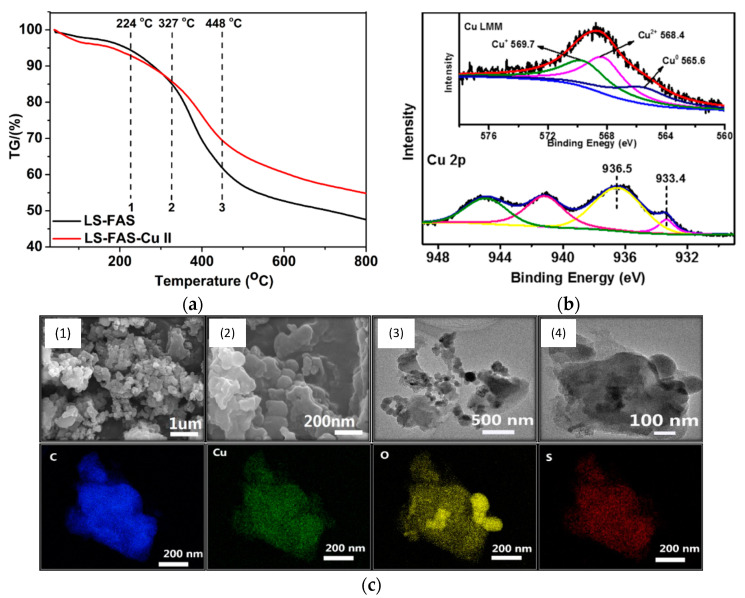
(**a**) Thermogravimetric analysis of the obtained **LSA-FAS** and **LSA-FAS-Cu^2+^**. (**b**) XPS spectra of **LSA-FAS-Cu^2+^** in the Cu 2p_3/2_ and Cu LMM regions (inset). (**c**) FSEM images of **LSA-FAS-Cu^2+^** at different scales (top) and its elemental mapping showing C, Cu, O, and S elements (bottom). Adapted with permission from reference [33]. Copyright 2014 Atlanta Publishing House LLC.

Moreover, FTIR spectra of **LSA**, **LSA-FAS**, and **LSA-FAS-Cu^2+^** also revealed important pattern changes when compared to each other, where authors attributed this variation first to the incorporation of **FAS** units, and then by the complexation of Cu^2+^ species. Furthermore, the thermal stability of these materials was evaluated by thermogravimetric analysis (TGA), showing that **LSA-FAS** and **LSA-FAS-Cu^2+^**, despite subtle differences, exhibited outstanding thermal resistance when considering the temperature conditions usually employed in catalyzed organic transformations (Figure 5a). Complementing the above, the authors also assigned the different weight losses observed in the thermograms to certain degradation processes, such as moisture loss, elimination of SO_3_^−^ species, and finally the volatilization of organic fragments resulting from the main degradation of the polymeric material [37,38,39]. Then, the authors studied the catalyst through X-ray photoelectron spectroscopy (XPS), identifying C, O, F, S, and Cu as the main elements present in the chemical composition of the material’s surface, as was already expected considering the elements present in **LSA-FAS** and Cu(OTf)_2_. Interestingly, thanks to the high-resolution spectrum of Cu 2p accompanied by the analysis of the Auger Cu LMM spectrum, the coexistence of Cu^2+^, Cu^+^, and Cu^0^ species was confirmed, demonstrating that part of the Cu^2+^ precursor was partially reduced during catalyst preparation [40,41,42] (Figure 5b). Additionally, through field emission scanning electron microscopy (FESEM) and field emission transmission electron microscopy (FETEM), it was possible to observe the surface morphology of **LSA-FAS-Cu^2+^**, showing specimens with a granular/blocky morphology with sizes ranging from 100 to 400 nm. Finally, elemental mapping images showed a uniform distribution of C, O, S, and Cu in **LSA-FAS-Cu^2+^** (Figure 5c).

### 2.3. Catalysts Based on LSA as a Support Matrix for the Immobilization of Metal Nanoparticles (LSA-MNPs)

The good results achieved by **LSA-M^n+^** materials in the catalytic formation of C–C bonds served as motivation to continue developing new lignin-based systems for this purpose. In this sense, it was a matter of time for nanotechnology to reach this topic, supported by the outstanding performance that metal nanoparticles (MNPs), of diverse nature, have been shown in a large variety of catalytic transformations [43,44,45]. It seems that, unintentionally, the development of **LSA-M^n+^** catalysts paved the way to prepare lignin-based nanocomposites containing MNPs due to the easy access to this type of entity from the already impregnated ionic metal species in **LSA-M^n+^**. Indeed, by subjecting **LSA-M^n+^** specimens to a reducing step, **LSA-MNP** nanocomposites could be achieved. Notwithstanding the above, another option for the preparation of these materials is by directly incorporating previously synthesized nanoparticles into the lignin matrix, a method that is usually referred to as “wet impregnation” [46,47]. Regarding the reducing step, there are a large variety of suitable reducing agents that have been successfully tested in the formation of metal nanoparticles [48]; however, a high interest has emerged in employing protocols where external reducing agents can be replaced by temperature, avoiding the excessive implication of chemical substances, and thereby offering a greener methodology [49]. On the other hand, the lignin matrix plays a significant role during the synthesis of MNPs, not just serving as mere support but also being implicated in the stabilization of these nanostructures, controlling both nucleation and growth mechanisms aiming to ideally prevent the appearance of agglomerates or obtain structures exceeding the nanometric dimension. Surprisingly, even though the first report about using lignin as an MNP support and its catalytic assessment for the construction of C–C bonds was more than a decade ago, to date, the literature related to this is still scarce. Indeed, the first report came out in 2009 by Guillén and coworkers [50]; however, it must be mentioned that in this case, lignin was used as an activated carbon precursor and not in its pristine form. The authors first carried out the impregnation of lignin with H_3_PO_4_ followed by its activation under an N_2_ atmosphere using a conventional tubular furnace at 500 °C for 2 h. After being cooled under an inert atmosphere, the sample was thoroughly rinsed with warm water (60 °C) until neutral pH and there was a lack of phosphate traces in the washing waters. The obtained material was dried at 100 °C, yielding an activated carbon material labeled **LAC**. Then, a portion of **LAC** was submitted to an additional thermal program consisting of heating the sample up to 900 °C, also under an N_2_ atmosphere, achieving the preparation of a second carbonaceous material named **LACT**. Both materials were subsequently used as support matrices of palladium nanoparticles (PdNPs), which were incorporated through wet impregnation. Regarding the above, **LAC** and **LACT** were introduced into an aqueous solution of PdCl_2_ with a concentration fixed to reach a metal loading of approximately 0.5 wt%. Finally, the impregnated palladium ions on both supports were reduced at 400 °C using a mixed N_2_/H_2_ gaseous atmosphere (ratio 3:1), affording **LAC-Pd** and **LACT-Pd** catalysts. The textural properties of both catalysts were studied by N_2_ adsorption–desorption isotherms performed at low temperatures (−196 °C) and with CO_2_ at 0 °C, from which a very complete and comprehensive characterization of the porosity of samples was achieved. In this sense, parameters such as the apparent surface area, the external surface area (associated with the nonmicroporous structure), the micropore volume, and the specific surface area were calculated employing well-known fitting models [51,52]. From the above results, the authors stated that both materials presented a heterogeneous porous structure characterized by a wide microporous structure but also a well-developed mesoporosity. Moreover, through Brunauer–Emmett–Teller (BET) analysis, surface areas for all obtained materials (with and without PdNPs) were calculated. Interestingly, this last parameter seems to be highly affected in those samples pyrolyzed at 900 °C, where lower values were measured for **LACT** compared to **LAC**. The authors explained this based on a possible thermal-triggered reorganization experienced by these materials at high temperatures, promoting the shrinking of their porous structure [53]. On the other hand, even lower surface areas were determined for samples decorated with PdNPs, ascribed to the volume occupation that these entities exert after being generated within the cavities of the material. Therefore, the employment of phosphoric acid as an activating agent is a good strategy to afford an adequate porosity, favoring the crosslinking process between different portions of the polymer through the formation of polyphosphate bridges that are eventually removed during the washing step [54,55]. The authors also characterized both carbonaceous matrices and their corresponding Pd-containing catalysts by XPS. Thanks to this technique, they were able to corroborate the expected surface elemental composition of these materials, evidencing the presence of C, O, P, and N in **LAC** and **LACT,** while Pd was also detected in **LAC-Pd** and **LACT-Pd** catalysts. The mass percentage of Pd was very close to the targeted nominal value of 0.43% and 0.50% for **LAC-Pd** and **LACT-Pd**, respectively. The authors explained the presence of the unexpected nitrogen content as part of the initial composition for the Kraft lignin used as a precursor, while the phosphorous content was related to traces that were not removed after the washing step. A more in-depth XPS analysis allowed, in the case of P, verification of its pentavalent tetracoordinated nature, accusing the presence of species such as phosphates and polyphosphates that after the thermal treatment become part of the carbonaceous material [56,57]. More specifically, the deconvolution analysis for the P band allowed the identification of phosphorus-containing fragments with different chemical surroundings, for instance, C–O–PO_3_, C–PO_3_, and C_3_P [58,59,60]. Surprisingly, the **LACT** sample seems to have a richer composition in C–PO_3_ and C_3_P species than **LAC**, suggesting that thermal treatment has a high impact on this result. Similarly, the authors carried out a study on the Pd content and found that both Pd^0^ and Pd^2+^ species were present in samples **LAC-Pd** and **LACT-Pd**. However, the Pd^0^ content was always higher (71.7% vs. 28.3% and 59.8% vs. 40.2% for **LAC-Pd** and **LACT-Pd**, respectively). Finally, by TEM analysis, the authors were able to visualize PdNPs exhibiting sizes of approximately 5 nm together with a proper distribution along the catalyst surface.

A year later, the same research group extended this protocol for preparing mesoporous-activated carbons using lignin as the carbon source [61]. However, unlike their previous work, in this opportunity, the authors tested and compared the catalytic activity of samples in which Pd impregnation was carried out before or after thermal treatment at 900 °C. For clarity, the catalyst obtained after the H_3_PO_4_ activation stage at 500 °C was labeled **L**, and after palladium impregnation/reduction, it was labeled **L-Pd**. On the other hand, starting from **L**, two different types of samples were achieved: (1) those named **LT-Pd** resulting from, first, the heating of **L** at 900 °C followed by the impregnation/reducing step of Pd precursors and (2) those labeled **L-PdT** in which the impregnation/reducing step of Pd was carried out prior to the pyrolysis process. All materials were subjected to the same type of characterization performed in their first work, observing similar trends. For instance, gas adsorption–desorption isotherms revealed that those samples exposed at 900 °C showed, as expected, a decrease in their surface areas, which was even lower in the presence of palladium. However, a strong point of this report relies on the study of the Pd chemical state dependence when metal impregnation was carried out prior to or after the calcination step. In this sense, through XPS analysis, Pd 3d_5/2_ and Pd 3d_3/2_ signals indicated the coexistence of both Pd^0^ and Pd^2+^ species in all catalysts [62,63,64]. Moreover, the deconvolution of the Pd 3d region in the obtained spectra allowed calculating the Pd^0^/Pd^2+^ ratio and how this value varies depending on the thermal treatment applied to the samples. Surprisingly, the **L-Pd** and **LT-Pd** catalysts showed a Pd content richer in zero-valent species with percentages of approximately 71.7% and 59.8%, respectively. Conversely, **L-PdT** samples exhibited a remarkably higher proportion of Pd^2+^ species (77.1%), showing that, apparently, during the pyrolysis stage, an important fraction of the metallic Pd is oxidized. The authors argued that a feasible explanation for the above could be the oxidation of Pd^0^ triggered by the reduction of C–O–PO_3_ groups to C–PO_3_ and C_3_PO entities. Additionally, from TGA measurements, it was evidenced that at 900 °C, most of the lignin precursor is decomposed, and due to its chemical structure, it is highly possible to expect CO_2_ evolution as a degradation product. Therefore, the authors also proposed that CO_2_ could be dissociated on Pd nanoparticle surfaces, generating highly oxidative species that would promote the transformation of Pd^0^ into Pd^2+^ [65,66]. Finally, palladium particles present in the **L-Pd**, **LT-Pd**, and **L-PdT** catalysts were visualized by TEM and analyzed in terms of their size and size distributions. **L-Pd** showed a narrower size distribution with entities between 3 and 6 nm and an average value of 4.5 nm. Moreover, this sample stood out by the high dispersion exhibited by PdNPs across the material, ascribed to the high acidity of the carbon support due to the H_3_PO_4_ activation process that lowers the hydrophobicity of the system [59,67]. In contrast, both samples subjected to the thermal treatment showed broader size distributions and larger average particle sizes (11.7 nm and 9.7 nm for **L-PdT** and **LT-Pd**, respectively), ascribed to sintering processes that metal nanoparticles could experience under these conditions [68]. Lastly, the authors also confirmed the oxidant resistance of these catalysts by exposing them to thermogravimetric analysis under air conditions. This was explored motivated by the already detected P content in the samples, since it has been well-reported that phosphorus can act as an inhibitor of the oxidation of carbon materials [69,70]. As a result, all catalysts showed high oxidation resistance by showing no significant decomposition below 450 °C, allowing us to expand the experimental conditions in which these materials can be used as catalysts.

**Figure 6:**
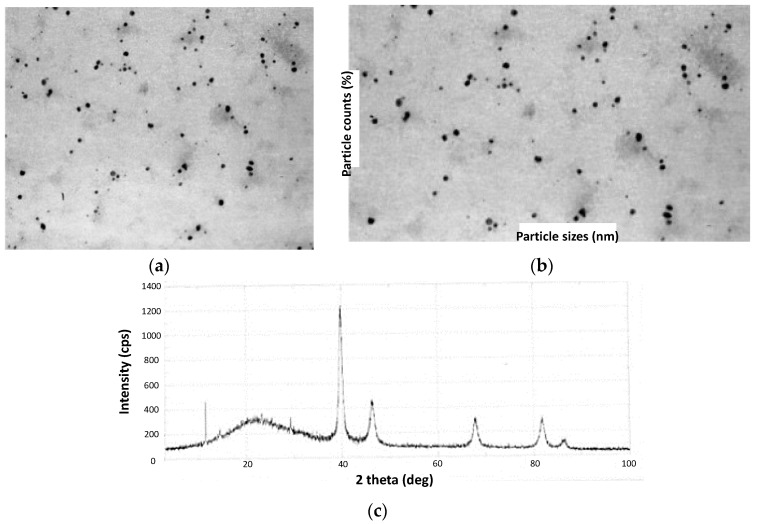
(**a**) TEM image of Kraft lignin PdNPs and (**b**) its statistical distribution of NP dimensions. (**c**) XRD pattern of Kraft lignin PdNPs. Adapted with permission from reference [71]. Copyright 2013 Elsevier B.V.

In 2013, Coccia and coworkers prepared PdNPs using water-soluble lignin as a reducing and stabilizing agent [71]. This can be considered one of the first reports on using lignin in its pristine form for the elaboration of catalysts for C–C bond formation and not as a mere source of active carbonaceous systems. The method stood out by its green experimental conditions in terms of moderate temperatures, short reaction times, and the use of aerated water as the reaction medium. They employed two types of lignin, the first corresponding to Kraft lignin, while the second was a lignosulfonated sample in the form of an ammonium derivative (**LSA-NH_4_^+^**). This last lignin was obtained from red pine andwas reacted in a calcium hydrogen sulfite solution. Lignins were codissolved with PdCl_2_ in water and heated at 80 °C for 3 h, after which a color change from brown to black was observed. The authors followed the reaction course by ultraviolet-visible (UV-Vis) spectroscopy, determining subtle changes in the visible region of the spectra accusing the formation of PdNPs, which were finally visualized through TEM analysis (Figure 6a). Spherical nanoparticles were obtained with both lignin materials, with a slight tendency toward agglomeration. However, UV-Vis analysis over time demonstrated good stability by not detecting relevant changes in the spectra even after a month of storage under aerobic conditions and room temperature (RT). Interestingly, PdNP sizes were highly influenced by the type of lignin used. In this regard, when using **LSA-NH_4_^+^**, values between 16 and 20 nm were achieved, while the use of Kraft lignin allowed us to reach values in the range of 8–14 nm (Figure 6b). On the other hand, the obtained lignin/PdNP catalysts in powder form were also characterized by X-ray diffraction (XRD), revealing a clear crystalline structure with peaks centered at 39.9°, 46.3°, 67.4°, 82.5°, and 86.9° attributed to PdNPs (Figure 6c) [72,73]. The authors also evidenced a thinner structure for these peaks in the sample prepared from Kraft lignin, as was expected due to the lower particle size. After confirming the presence of PdNPs, the authors carried out proton nuclear magnetic resonance (^1^H-NMR) and FTIR experiments aiming to investigate possible changes suffered by lignin after the reducing step. Surprisingly, ^1^H-NMR spectra signals ascribed to small amounts of acetic acid and formic acid, as well as methanol, were found that, based on the author’s arguments, could be assigned to oxidation products coming from the polymer backbone. On the other hand, FTIR spectra revealed that most of the vibrational bands ascribed to pristine lignin remained unchanged, with the exception of a new weak signal centered at 1730 cm^−1^ that could be related to carbonyl species that would emerge from, for example, the oxidation of alcoholic residues that participate during Pd^+2^ reduction [74].

Three years later, in 2016, another report dealing with the deposition of PdNPs on lignin (**LSA-PdNPs**) and its catalytic evaluation in Mizoroki–Heck couplings was performed by Marulasiddeshwara and Kumar [61]. Here, the authors synthesized PdNPs from PdCl_2_ aqueous solutions in the presence of lignin. To achieve this, two strategies were tested: (1) mixing PdCl_2_ and lignin in water at an ambient temperature, followed by the addition of hydrazine monohydrate as a reducing agent and (2) mixing PdCl_2_ and lignin in water followed by boiling. In the latter case, lignin serves both as a reducing and stabilizing agent. Nevertheless, both strategies showed similar qualitative behavior, in which red solutions ascribed to the presence of the PdCl_2_ complex turned dark gray due to the formation of PdNPs. Indeed, a good option for monitoring the reaction was through UV-Vis analysis, from which the band’s disappearance centered at 430 nm indicates the consumption of PdCl_2_ [75,76,77]. After completing the reaction time according to each strategy, catalysts were isolated by removing the solvent under reduced pressure, washing the gray residue, and ending with a drying step in a vacuum oven. The obtained specimens turned out to be insoluble in conventional organic solvents, promoting their use as heterogeneous catalysts. However, a series of advantages were found for strategy two, such as better stability against aggregation phenomena, since a more intimate interaction between PdNPs and lignin is achieved by the dual function that this biopolymer exerts in this case. Therefore, initially, the coordination of palladium cations on the polymer backbone is expected, followed by their reduction by the lignin’s functional groups. Additionally, by high-resolution ICP-AES analysis, it was found that the amount of loaded Pd was higher using this strategy, being approximately 0.0467 mmol/g. Based on the above arguments, the authors centered the catalytic evaluation using the sample achieved by strategy two. TEM images allowed the visualization of PdNPs impregnated on the lignin matrix, from which spherical entities were identified with sizes between 1 and 5 nm. As a complement, SEM allowed the authors to detect a proper dispersion of these nanoentities over the lignin surface, also finding a good correlation with the size measured by TEM. Furthermore, energy dispersive X-ray (EDX) analysis confirmed the elemental composition of the observed nanostructures. The catalyst powder was subjected to XRD analysis, revealing the presence of sharp peaks that corroborated the high crystallinity of the obtained PdNPs characterized by an fcc structure [78,79]. Finally, some insights into the role of lignin as a reducing agent during nanoparticle synthesis were identified through FTIR analysis. In this regard, the FTIR spectra of pure lignin and the catalyst showed a very similar pattern with subtle differences that could be related to the involvement of lignin during the reducing step and the presence of interactions between PdNPs and the lignin structure. Therefore, the participation of this biopolymer in the reduction of Pd^2+^ would be supported by the appearance of a new vibrational band centered at 1703 cm^−1^, typically observed for carbonyl species formed after the oxidation of, for instance, hydroxyl groups. In addition, lignin/PdNP interactions could be associated with the redshift observed for the O–H band after nanoparticle incorporation.

**Figure 7:**
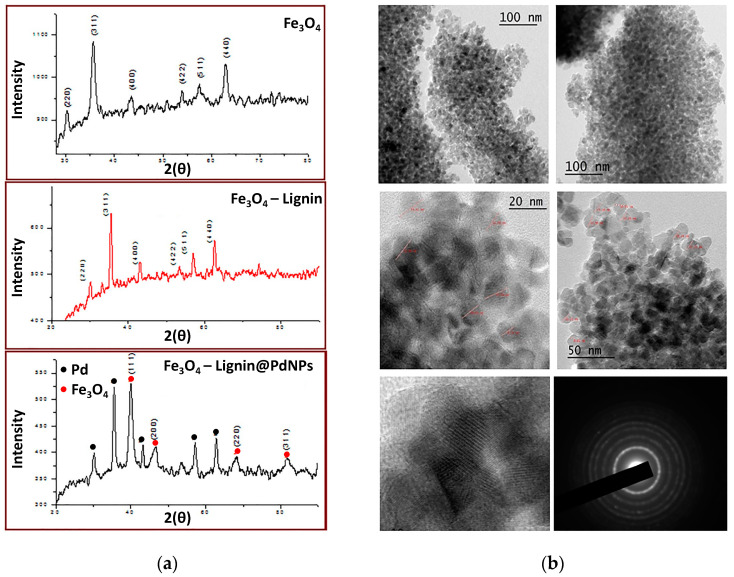
(**a**) XRD spectra of Fe_3_O_4_, **LSA-Fe_3_O_4_NPs**, and **LSA-Fe_3_O_4_NPs-PdNPs**. (**b**) TEM images at different scales for the obtained **LSA-Fe_3_O_4_NPs-PdNPs** catalyst. Adapted with permission from reference [80]. Copyright 2020 John Wiley and Sons Ltd.

Later, in 2020, Marulasiddeshwara and Kumar contributed again to the development of a palladium-containing lignin catalyst, but in this opportunity, the sample was endowed with a magnetic response due to the presence of Fe_3_O_4_ nanoparticles (Fe_3_O_4_NPs) [80]. The synthesis of the catalyst started by preparing Fe_3_O_4_ nanoparticles through chemical coprecipitation employing FeCl_3_ and FeSO_4_ in a 2:1 ratio as sources of Fe^3+^ and Fe^2+^, respectively [81]. Both salts were dissolved in water and heated to 85 °C under constant stirring and an inert atmosphere. Then, a NaOH aqueous solution was added to achieve the formation of the Fe_3_O_4_NPs in the form of a black precipitate, which was washed thoroughly with water and dried. Subsequently, the hybrid **LSA-Fe_3_O_4_NP** system was prepared by first dispersing certain aTmounts of Fe_3_O_4_NPs through sonication in an ethanol/water mixture (1:1) followed by the addition of lignin. The mixture was maintained for 5 h at 40 °C, and the solid was extracted by magnetic decantation. Finally, the preparation of the catalyst was carried out by adding a PdCl_2_ solution dropwise over an aqueous dispersion of **LSA-Fe_3_O_4_NPs** to then incorporate a NaOH solution. The above mixture was heated under reflux for 6 h, after which the reaction mixture turned black. Catalyst purification was achieved through magnetic decantation, meticulous washing steps, and a final drying stage at 100 °C in a vacuum oven. Similar to their previous work, the consumption of PdCl_2_ was confirmed by the vanishing of the band centered at 430 nm in the UV-Vis spectrum, indicating the formation of Pd^0^ species. On the other hand, in addition to signals belonging to lignin, FTIR analysis allowed the identification of signals at 574 and 572 cm^−1^ attributed to Fe–O vibrations, corroborating the presence of Fe_3_O_4_NPs [82,83,84]. Then, **LSA-Fe_3_O_4_NPs** and **LSA-Fe_3_O_4_NPs-PdNPs** were analyzed by XRD, corroborating the simultaneous presence of both Fe_3_O_4_NP and PdNP species thanks to the characteristic peaks observed in the diffraction patterns, also demonstrating their crystalline structure (Figure 7a). The authors estimated a nanoparticle size between 5 and 10 nm from the XRD data using the Debye–Scherer equation. On the other hand, they calculated an average size of 20 nm for Fe_3_O_4_NPs by TEM analysis (Figure 7b). With the aim of confirming the presence of both types of nanostructures, the authors resorted to EDX measurements through which Pd and Fe were successfully detected, while the quantification of both elements was measured by means of HRICP-AES, reaching values of 11.88 and 10.90 wt%, respectively, per gram of catalyst. The inclusion of nanostructures with a magnetic response, such as Fe_3_O_4_NPs, could open the possibility of achieving a more facile recovery of the catalytic system, prolonging its useful life.

## 3. Lignin-Based Catalysts for C–C Bond Formation

In this section, we will discuss the use of the above-described **LSA**, **LSA-M^n+^**, and **LSA-MNPs** as solid heterogeneous catalysts for different C–C bond-forming reactions. Although the first use involving **LSA**-based catalysts for C–C bond formation was approximately 14 years ago [50], this specific field is still in its infancy according to the total number of contributions in the literature and the variety of reactions tested thus far.

### 3.1. LSA-Catalyzed C–C Bond-Forming Reactions

#### 3.1.1. LSA

1. Multi-Component Reactions (MCR)

**Scheme 1:**
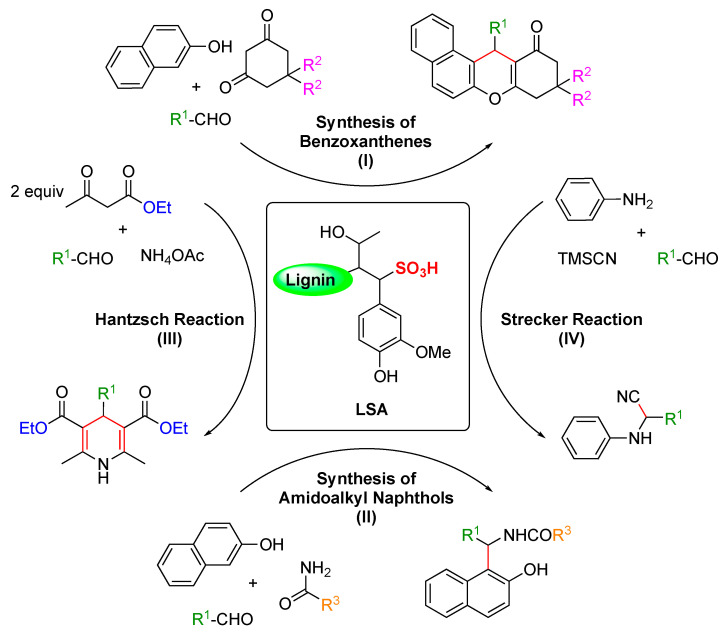
**LSA**-catalyzed C–C bond formation in various MCRs [31].

Multi-component reactions (MCRs) enable the construction of complex organic molecules in one pot with high atom economy and high selectivity [85,86]. When involving aldehydes, acid catalysts such as InCl_3_, Sr(OTf)_2_, ceric ammonium nitrate (CAN), proline, *p*-toluene sulfonic acid (*p*-TSA), NaHSO_4_·SiO_2_, and ionic liquids have been frequently used to activate the aldehyde component, enabling the nucleophiles to react easily [87]. Nevertheless, some of these procedures sometimes use toxic organic solvents, harsh reaction conditions, and low yields. Within this context, Chen and coworkers reported in 2015 the use of **LSA** as a solid acid catalyst for some important multi-component reactions, such as the synthesis of benzoxanthenes and amidoalkyl naphthols, the Hantzsch reaction, and the Strecker reaction (Scheme 1, I) [31]. The reactions were carried out in one pot without solvent at RT or 90 °C for 1–2 h using **LSA** with a concentration of −SO_3_H of 1.88 mmol g^−1^ (corresponding to ca. 6.0% (*w*/*w*) sulfur content). In the case of the synthesis of benzoxanthenes, optimization experiments revealed 3.8 mol% **LSA** as the optimal concentration.

Thus, a variety of aromatic aldehydes bearing electron-donating or electron-withdrawing groups afforded the desired products in very good yields (82–93%) (Table 1, entries 1–8). A comparison with other catalysts previously reported in the literature for this transformation (i.e., *p*-TSA, Sr(OTf)_2_, InCl_3_, CAN, praline triflate, and immobilized ionic liquids) showed a similar catalytic performance of **LSA** but with the advantages of low cost, biodegradability, and renewability. It is worth mentioning that benzoxanthene derivatives are remarkable synthons for the preparation of bioactive compounds [88].

Moreover, in the same publication [31], the authors demonstrated that **LSA** is also very effective in catalyzing the synthesis of 1-amidoalkyl-2-napthols (Scheme 1, II). These molecules belong to a family of compounds with recognized bioactivity [89,90] and are normally prepared using Lewis and Brønsted acids [91,92]. Thus, it was expected that **LSA** could also catalyze the same reactions. Indeed, a series of aromatic aldehydes bearing both electron-withdrawing and electron-donating groups yielded the corresponding products in excellent yields (84–94%) (Table 1, entries 9–16).

Finally, the same group successfully carried out two additional important reactions, namely, the Hantzch and Strecker reactions (Scheme 1, III, and IV, respectively) catalyzed by **LSA** [31]. The Hantzch reaction was performed by a condensation reaction of 4-methylbenzaldehyde, ethylacetoacetate, and ammonium acetate, affording the corresponding 1,4-dihydropyridine derivative in a 90% yield (Table 1, entry 17). Importantly, this class of compounds is also known to possess remarkable bioactivities [93,94]. Furthermore, the Strecker reaction between 4-methylbenzaldehyde, aniline, and TMSCN gave the desired α-amino nitrile derivative in a 92% yield (Table 1, entry 18).

Importantly, the **LSA** used in the previous transformations was found to be stable in terms of the molecular structure, crystallographic properties, and morphology during the reactions, as indicated by FT-IR, XRD, and SEM characterizations of the recovered solid catalyst [31]. In addition, **LSA** could be reused up to four times with a very minor loss of the initial catalytic activity due to a slight decrease in the −SO_3_H content to 1.79 mmol g^−1^.

2. Phenol–Aldehyde Condensation

**Scheme 2:**
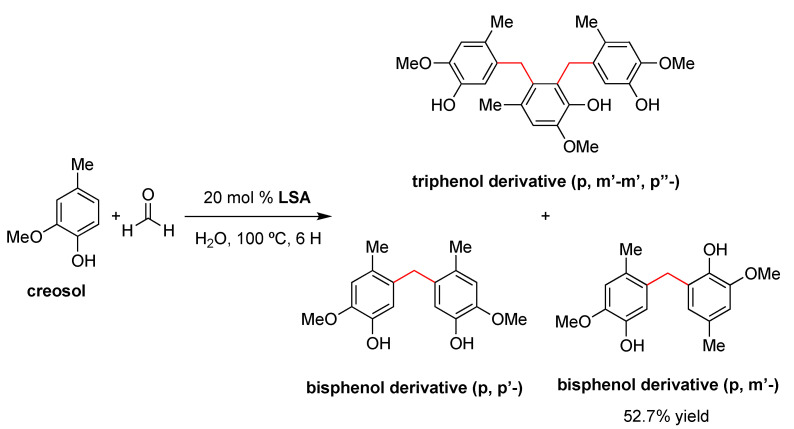
**LSA**-catalyzed condensation of creosol (2 equiv) and formaldehyde (1 equiv) in water [95].

In 2015, Chen and coworkers reported the use of **LSA** (with 7.3% sulfur content) as a catalyst for the preparation of bisphenols by a condensation reaction between two equivalents of creosol and formaldehyde in water [95]. This reaction is usually catalyzed by mineral acids such as H_2_SO_4_ or HCl [96], although these traditional methods have some disadvantages, such as the generation of hazardous waste and the difficult separation of the desired product among different isomers. For comparative purposes, the authors also performed condensation with traditional catalysts such as acetic acid (AcOH), HCl, H_2_SO_4,_ benzenesulfonic acid (BSA), and *p*-toluene sulfonic acid (*p*-TSA). The sequence of catalytic activity was found to be BSA > *p*-TSA > **LSA** > HCl > H_2_SO_4_ >> AcOH, affording the bisphenol derivative (isomer p,m’-) as the main product due to both steric and electronic effects [96]. The optimum conditions were found to be 20% mol of **LSA** at 100 °C for 6 h, providing the desired product in 52.7% yield (Scheme 2). Comparing the yields and pKa of the above-mentioned acids, the authors proposed that the pKa values of **LSA** may be close to those of BSA (pKa = 3.5), *p*-TSA (pKa = 1.99) and methyl sulfonic acid (pKa = −1.75) [97]. In any event, the acidity of the catalyst should be sufficient not only to activate the aldehydes but also to avoid side reactions [98]. In this sense, **LSA** was effective in promoting the condensation of creosol with formaldehyde but not with other aldehydes, such as acetaldehyde and propionaldehyde, presumably due to the electron-donating effect of the alkyl substituents on **LSA**.

Importantly, the products outlined in Scheme 2 precipitated out from the reaction mixture, facilitating its isolation by a simple protocol of filtration and washing with ethyl acetate. The remaining acidic solution was used in four subsequent reactions, affording the desired products in approximately the same yield. NMR, FTIR, and elemental analyses of the recycled **LSA** indicated some slight unidentified structural changes of the catalyst during the reaction, albeit they did not affect its catalytic performance [95]. In terms of the mechanism, the process seems to occur as with other acidic catalysts. Briefly, the initial protonation of formaldehyde by **LSA** in water is followed by electrophilic aromatic substitution at the *para*-position of creosol, affording the corresponding *p*-hydroxymethyl creosol derivative, which undergoes a series of consecutive protonation–dehydration–substitution events to afford the desired product [95].

#### 3.1.2. LSA-IL@NH_2_

1. Knoevenagel Condensation

**Scheme 3:**
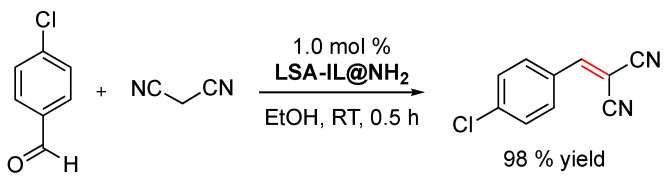
**LSA-IL@NH_2_**-promoted Knoevenagel condensation between 4-chlorobenzaldehyde and malononitrile (1.1 equiv) [33].

In the field of green processes, one of the advantages of using ionic liquids in chemistry is that, since they do not have a measurable partial pressure, they do not emit volatile organic compounds (VOCs), unlike conventional molecular liquids [99]. Moreover, the immobilization of ionic liquids on solid supports increases their recyclability. In this context, Sun and coworkers demonstrated that an amino-functionalized imidazolium-based ionic liquid immobilized on ammonium lignosulfonate (i.e., **LSA-IL@NH_2_**) via an ion-exchange procedure constitutes an effective catalyst to promote the Knoevenagel condensation between 4-chlorobenzaldehyde and malonitrile (Scheme 3) [33]. Furthermore, **LSA-IL@NH_2_** afforded the desired α,β-unsaturated ketone in a 98% yield, a much higher value than that of another commercially available biocatalyst such as chitosan (27% yield) under comparable experimental conditions [33]. Taking into consideration that the Knoevenagel reaction is usually catalyzed by weakly basic amines [100], the increase in product yield observed with **LSA-IL@NH_2_** seems to be related to larger accessibility of the substrate to the -NH_2_ groups of the catalyst compared to chitosan. In terms of reusability, **LSA-IL@NH_2_** could be reused at least three times without significant loss in the catalytic activity (i.e., product yield after three cycles = 97%).

### 3.2. LSA-M^n+^-Catalyzed Reactions

#### 3.2.1. LSA-Sc^3+^

1. Michael addition/dehydration tandem reactions

In a comprehensive study, Sun and coworkers described a model Michael/addition dehydration tandem reaction between 3-acetyl-2-hydroxy-2-methylchromenes and indoles catalyzed by **LSA-Sc^3+^** (0.44 mmol g^−1^) in EtOH [33] (Table 2). This reaction enabled the preparation of highly substituted 4*H*-chromenes in good and excellent yields (61–93%). Other acid catalysts, such as *p*-TSA, also afforded the desired compounds under comparable conditions, albeit with lower yields. Preliminary optimization experiments in general fixed the catalyst amount, temperature, and reaction time at 1.2 mol%, 80 °C, and 8 h, respectively. The use of **LSA-Sc^3+^** with lower Sc^3+^ loading (0.22 mmol g^−1^) yielded the desired product, albeit in a much lower yield. Furthermore, control experiments in the absence of the catalyst and with **LSA-Na^+^** demonstrated that the immobilized Sc^3+^ is critical in this process. Although electron-rich and electron-poor indoles afforded the desired compounds, indoles bearing a strongly electron-withdrawing group, such as carboxyl, were unreactive. Importantly, the solid catalyst was isolated by centrifugation after one of the reactions, and the solution was allowed to react again during the same period of time. The result showed essentially no variation in the product yield, indicating no leaching of Sc^3+^ into the reaction mixture. The stability of the metal content of the catalyst was also confirmed by ICP-MS measurements. In addition, **LSA-Sc^3+^** was recovered after the reaction, washed with EtOH, dried under vacuum at 60 °C, and subject to the next run. After three cycles, the product yield remained almost constant with only a slight decrease (Table 2, entry 1), presumably due to a minor loss of the catalyst during the filtration and washing protocols. However, other solid-supported scandium catalysts, such as **SiO_2_-Sc^3+^** and **resin-Sc^3+,^** showed a significant reduction in the product yield in the third run as well as a reduction (Table 2, entries 15–16 vs. entry 1) in the metal loading, suggesting a more effective immobilization of the metal ion onto **LSA** with a high density of hydroxy and carboxyl groups. Furthermore, **LSA-Sc^3+^** also enabled the reaction to be carried out on a larger scale (10 mmol) without decreasing the product yield. This tandem reaction was also carried out using antipyrine as a nucleophile instead of an indole compound, affording the corresponding chromene derivative in good yield [33].

2. Electrophilic Ring-Opening Reactions

**LSA-Sc^3+^** was also found to be an efficient catalyst for electrophilic ring-opening of 2-butoxy-3,4-dihydropyran with indoles [33]. This reaction is commonly catalyzed by acids and is very sensitive to the strength of the acid. Some of the most common catalysts for this transformation include Mn(II) halides [101], although they have the disadvantage of being nonrecyclable. In contrast, **LSA-Sc^3+^** (0.44 mmol g^−1^) enabled the reaction to afford the desired product in very good yields (Table 3). In this case, preliminary experiments showed the optimal conditions for the use of 10 mol% catalyst and CH_3_NO_2_ as the solvent at 100 °C for 11 h. The use of nitromethane facilitates the recovery of the insoluble catalyst by simple filtration. In this study, **LSA-Sc^3+^** was used at least twice in a model reaction (Table 3, entry 1) without significant detriment to the product yield. In a valuable self-criticism, the authors pointed out that nitromethane is a toxic and explosive solvent. Thus, future replacement of this solvent for a greener alternative would be highly desired. In terms of the substrate scope, a variety of substituted 2-butoxy-3,4-dihydropyrans and indoles gave the corresponding adducts in yields ranging from 63% to 96% (Table 3). These results indicated a good tolerance of the process to esters and halides. It is worth mentioning that the use of Sc(OTf)_3_ as a catalyst afforded the desired product in the model reaction but in a much lower yield (23%).

3. C–H Alkylation of Indoles

**Scheme 4:**
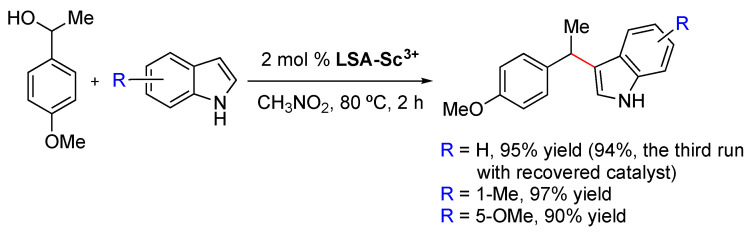
**LS-Sc^3+^** promoted the reaction of α-methyl-4-methoxybenzyl alcohol with indoles [33].

**LSA-Sc^3+^** (0.44 mmol g^−1^) has been used as an efficient solid catalyst for the reaction of α-methyl-4-methoxybenzyl alcohol and indoles, affording the corresponding 3-benzylindole derivatives in excellent yields (Scheme 4) [33]. In contrast to other commonly used acid catalysts, **LSA-Sc^3+^** could be easily recovered by filtration and reused at least twice without significant loss of its catalytic activity. Compared to **LSA-Sc^3+^**, the use of other acids such as triflic acid or sulfamic acid also afforded the desired products, albeit in lower yields (66% and 83% (after 8 h), respectively), and the catalysts cannot be reused.

#### 3.2.2. LSA-Cu^2+^

Another metal widely used in C–C bond-forming reactions is Cu^2+^. As explained in Section 2.2., Cu^2+^ can be easily immobilized on **LSA** using Cu(OTf)_2_ as the copper source. The obtained **LSA-Cu^2+^** (0.80 mmol g^−1^) was found to be highly active in several important reactions, which are summarized below.

1. Multi-Component Reactions (MCR)

As described in Section 2.2., Lai and coworkers [34] reported the condensation of **LSA** with **FAS** which increased the concentration of –SO_3_H groups and facilitated coordination with significantly higher amounts of Cu^2+^ ions compared to **LSA-Cu^2+^**. This afforded the obtention of the **LSA-FAS-Cu^2+^** catalyst with a metal loading of 0.92 mmol g^−1^ [34]. The use of 20 mol% of this catalyst was effective in the three-component reaction of 4-aminoindole, 4-methylbenzaldehyde, and diethyl acetylenedicarboxylate in EtOH at 60 °C (optimized conditions), affording a seven-membered indole tricyclic system in 86% yield (Table 4, entry 1). This type of molecular structure is interesting due to the biological activities described for various tricyclic indole alkaloids [102]. Importantly, the reaction could be scaled up to 10 mmol without significant detriment to the product yield (84%). In terms of the substrate scope, the reaction tolerated aldehydes with different functional groups on the aromatic ring, yielding the 3,4-fused tricyclic indoles in modest yields (46–64%; Table 4, entries 2–9) and indicating potential steric hindrance effects. Aliphatic aldehydes were also converted into the desired products in good to modest and good yields (45–80%; Table 4, entries 10–14). A significant yield reduction was observed when dimethyl acetylenedicarboxylate was used instead of the diethyl analog (Table 4, entry 15 vs. entry 1).

2. Glaser Coupling Reaction (Hay Coupling Modification)

**Scheme 5:**
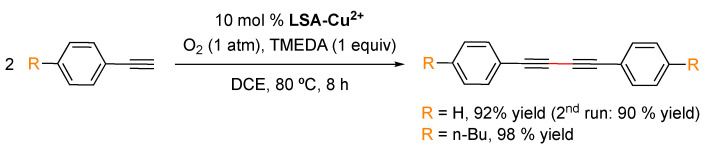
**LSA-Cu^2+^**-catalyzed Glaser reaction of phenylacetylenes [33].

The Hay coupling reaction is a modification of the Glaser coupling, which by far constitutes the oldest acetylenic coupling affording symmetric or cyclic bisacetylenes [103]. The reaction is based on the use of cuprous salts and an additional oxidant such as oxygen that reoxidizes the catalytic species in the reaction medium. The Hay coupling modification is more versatile due to the use of *N*,*N*,*N′*,*N′*-tetramethylethylenediamine (TMEDA) as the formed copper-TMEDA complex is soluble in a wider range of solvents, making the reaction more versatile. **LSA-Cu^2+^** has also been found to be an efficient and recyclable catalyst for the Glaser coupling reaction of phenyl acetylenes [33] (Scheme 5). This reaction was successfully performed in DCE as the solvent, where the catalyst is insoluble, at 80 °C for 8 h. Under these conditions, the expected products were isolated in more than a 90% yield. With the foregoing results, it is reasonable to think that other reactions catalyzed by copper(II) salts might also be catalyzed by **LSA-Cu^2+^**. It is worth mentioning that **LSA-Cu^2+^** has also been found to be very efficient for other important reactions involving C-heteroatom bond formation, such as the cycloaddition reaction between azides and alkynes [33].

3. Oxidative Coupling Reaction

**Scheme 6:**
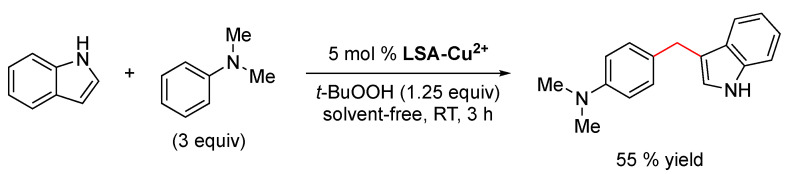
**LSA-Cu^2+^**-catalyzed oxidative coupling of *N*,*N*-dimethylaniline and indole [33].

The oxidative coupling of *N,N*-dimethylaniline and indole has also been performed using **LSA-Cu^2+^** as the catalyst, affording the coupled product in a 55% yield under solvent-free conditions at room temperature (Scheme 6). The reaction mechanism most likely involves the formation of formaldehyde via oxidation of the methyl group of *N*,*N*-dimethylaniline, followed by an electrophilic alkylation process [104]. Although 55% is a modest yield and not as good as the one obtained using ruthenium porphyrin (73% yield) [104], it should be considered that the latter is an expensive catalyst and more difficult to prepare. This transformation has been also carried out with sulfonic acid functionalized Fe_3_O_4_ nanoparticles under solvent-free conditions, obtaining the desired product in an excellent 87% yield in 1 h, albeit in this case the reaction was performed at 100 ºC without bio-based support [105].

4. Synthesis of 2-Arylpyridine Derivatives

Arylpyridines are important synthetic building blocks in different fields, such as biomedicine and functional materials [106,107]. Among the classical methods for the synthesis of arylpyridines (e.g., condensations, metathesis, cycloadditions, etc.) [108,109], a new methodology was recently reported using **LSA-FAS-Cu^2+^** acidified by H_2_SO_4_ (2 M) as a catalyst for the reaction of acetophenone and 1,3-diaminopropane in EtOH at 100 °C under aerobic conditions (optimized conditions), affording the corresponding 2-arylpyridine in a 74% yield (Table 5, entry 1). This yield was significantly reduced when a homogeneous CuOTf_2_ catalyst was used instead of the lignin-based catalyst. The reaction requires the use of 0.6 equiv of TsOH·H_2_O to obtain comparable results if the **LSA-FAS-Cu^2+^** is not previously acidified. In terms of the substrate scope, the reaction was found to tolerate different functionalities, such as halogens, cyclohexane, and benzyloxy moieties. Acetophenones bearing an electron-donating group in the *para*-position afforded the desired product in better yields than those with electron-withdrawing groups (Table 5, entries 2–10 vs. entries 11–13). Multisubstituted acetophenones and heterocycle-substituted ketones were also converted into the desired products in modest yields (Table 5, entries 16–21 and entries 22–23, respectively). Although the reactions proceeded smoothly, it should be noted the use of 40 mol% of the catalyst in these examplesis significantly higher than in other reactions catalyzed by **LSA-M^n+^** materials.

5. Synthesis of Aminonaphthalene Derivatives

**LSA-FAS-Cu^2+^** has also been used as a catalyst (10 mol%) for the synthesis of aminonaphthalenes from 2-(phenylethynyl)acetophenones and primary amines in DCE at 100 °C (Table 6). In terms of scope, *para*-substituted anilines with electron-donating groups afforded the desired products in better yields (Table 6, entries 1–3) than those bearing electron-withdrawing groups (Table 6, entries 4). Aliphatic amines and secondary amines were also converted satisfactorily (Table 6, entries 7–10). With respect to the ketone, F-, Cl-, aliphatic chain, and cycloolefin-substituted 2-(phenylethynyl)acetophenones reacted smoothly, providing the desired products in good yields (Table 6, entries 11–15). However, aniline derivatives bearing electron-withdrawing groups or acetophenone derivatives substituted with electron-donating groups were unreactive under these conditions (Table 6, entries 16–17).

### 3.3. LSA-MNPs

#### 3.3.1. LSA-PdNPs: Non-Carbonized Lignin-Based Catalyst

1. Suzuki–Miyaura Cross-Coupling Reaction

Suzuki–Miyaura reactions (also known simply as Suzuki reactions) involve C–C bond formation via the cross-coupling of organohalides and boronic acids catalyzed by a Pd(0)-complex. This process is widely used to synthesize styrenes, polyolefins, and substituted biaryls [110,111] both in organic solvents and in water [112,113,114]. Despite its versatility, this reaction also faces some challenges, such as the availability, stability, and cost of both Pd complexes and phenylphosphine ligands as well as the tedious separation procedure of traditional homogenous Pd catalysts. Within this context, in 2013 Coccia and coworkers reported the use of **LSA-PdNPs** (diameter from 10–20 nm, see Section 2.2.) to catalyze the Suzuki reaction between phenylboronic acid or p-hydroxymethyl phenylboronic acid and aryl halides under aqueous alkaline conditions (K_2_CO_3_) and aerobic conditions [71]. The optimum temperature (70 °C or 20 °C) and the reaction time were found to be substrate-dependent (Table 7). The optimum aryl halide:catalyst molar ratio was found to be 450:1 and control experiments in the absence of PdNPs showed no conversion. As shown in Table 7, the reactivity of aryl halides was found to follow the order iodo- > bromo- > chloro-derivatives. In terms of conversion and selectivity (i.e., homocoupling vs heterocoupling product), while both iodo- and bromobenzenes were almost quantitatively converted, the bromo derivatives did not always show a high selectivity, in some cases obtaining only 20% of the cross-coupling product vs 90% of conversion (Table 7, entry 9). At 20 °C, the iodo derivatives were found to be the most reactive substrates, selectively affording the desired cross-coupling products (Table 7, entries 2 and 11). In contrast, bromo derivatives at 20 °C yielded only 10–25% of the cross-coupling product (Table 7, entries 8, 10, 14). The low yield associated with 4-amino-iodobenzene (Table 7, entry 5) is due to its low solubility in alkaline water solution as well as partial polymerization processes taking place at 70 °C. Moreover, the effect of the base was tested using different bases, such as NaOH, NaHCO_3_, Et_3_N, and K_2_CO_3_. The results showed full conversion and selectivity only in the case of NaOH and K_2_CO_3_. Additional experiments demonstrated that the size of NPs and the electronic density on the aromatic ring were not critical factors for the reaction outcome [71].

2. Sonogashira Cross-Coupling Reaction

**Scheme 7:**
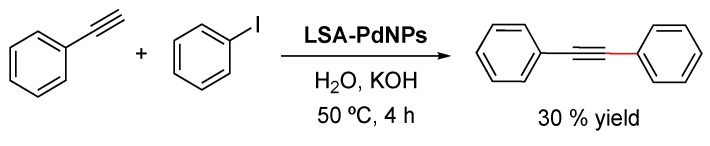
**LSA**-PdNP-catalyzed Sonogashira reaction between phenylacetylene (0.5 mmol) and iodobenzene (0.5 mmol) in an alkaline medium (water = 5 mL, KOH = 2 mmol). Reagent: **LSA-PdNPs** = 450:1 (molar ratio) [71].

The Sonogashira cross-coupling reaction (also called the Sonogashira–Hagihara reaction) consists of the coupling of terminal alkynes with an aryl or vinyl halide to form a C–C bond. The reaction commonly employs a Pd(0) catalyst, a Cu(I) cocatalyst, and a base to generate conjugated enzymes and arylalkynes [115,116] which have found applications in pharmaceuticals, natural products, organic materials, and nanomaterials [117]. The same **LSA-PdNPs** used for the Suzuki–Miyaura reaction also catalyzed the Sonogashira reaction of iodobenzene and phenylacetylene (Scheme 7) [71]. Although the reaction proceeded in the presence of KOH without the addition of CuI, phosphine, or amine ligands, the yield of the desired cross-coupled product was only 30%. Unfortunately, neither chlorobenzene nor bromobenzene reacted, making the methodology rather limited. It is worth mentioning that when CuI was added (5 mol% with respect to the aryl halide) to the reaction, only the alkyne dimer was formed, which represents the main side reaction of the Sonogashira reaction [117].

3. Stille Coupling Reaction

**Scheme 8:**
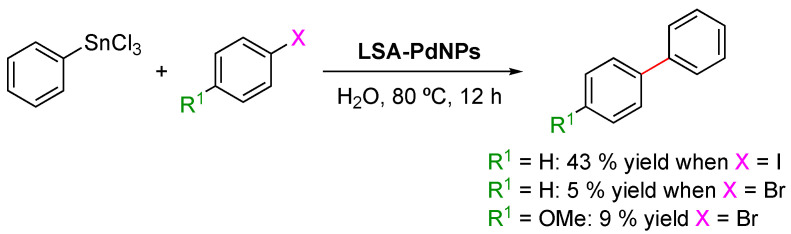
**LSA**-PdNP-catalyzed Stille reactions between trichorophenylstannane (0.75 mmol) and aryl halides (0.5 mmol) in water (5 mL) in the presence of K_2_CO_3_ (1.75 mmol). Reagent: **LSA-PdNPs** = 450:1 (molar ratio) [71].

The Stille reaction is a palladium(0)-catalyzed cross-coupling reaction widely used in organic synthesis, in which an aryl-, alkenyl-, or alkynyl-stannanne is cross-coupled with a vinyl or aryl halide, pseudohalide, or arenediazonium salt [118]. One of the biggest advantages of this reaction is its tolerance to most functional groups, making it an attractive methodology for the transformations of highly functionalized molecules of biological and pharmaceutical interest. Coccia and coworkers reported the Stille reaction between trichloro(phenyl)stannane and aryl halides (1.25:1 molar ratio), catalyzed by **LSA-PdNPs**, in the presence of K_2_CO_3_ in water at 80 °C [71] (Scheme 8). Similarly to the results for the Sonogashira coupling, the substrate scope was quite limited. Bromo-derivatives were poorly converted (5–9% yield), while iodobenzene afforded the desired cross-coupling product in a modest yield of 43% (Scheme 8).

4. Heck Cross-Coupling Reaction

The Heck reaction (also known as the Mizoroki–Heck reaction) is the coupling of unsaturated halides (or triflates) with alkenes in the presence of a base and a palladium catalyst to afford a substituted alkene via C(sp^2^)–C(sp^2^) bond formation [119]. Although this vinylation of aryl halides commonly uses iodine derivatives, it has been found that the use of phosphine ligands increases the reactivity of the aryl halides, facilitating the reaction with bromo derivatives. **LSA-PdNPs** have also been described as an efficient catalyst for this transformation in the presence of K_2_CO_3_ in water at 100 °C for 12 h (optimized conditions) (Table 8) [71]. It should be noted that the base is involved in the β-hydride elimination step, which speeds up the regeneration of Pd^0^ [120,121]. As expected, iodo derivatives showed higher reactivity than bromo analogs (Table 8, entry 1 vs entry 4), while chloro derivatives remained unreactive. The reaction between iodobenzene and styrene provided quantitative conversion with 100% selectivity in the coupling product (Table 8, entry 1). Aryl halides bearing -NH_2_ or -CO_2_H groups were also converted, albeit with lower yield and selectivity (Table 8, entries 2–3). In the case of aliphatic alkenes such as allyl alcohol, the reaction also afforded the desired product in a 30% yield, albeit requiring the use of a large excess of allyl alcohol to avoid the formation of biphenyl [71]. In this study, it was also concluded that the size of the NPs showed no major effect on the product yield, as was observed for Sonogashira coupling (see above).

In 2016, Marulasiddeshwara and Kumar reported the use of **LSA-PdNPs** to catalyze the reaction of haloarenes and substituted haloarenes with *n*-butyl acrylate [61] (Table 9). Preliminary experiments involving different solvents, bases, and reaction temperatures enabled us to set up the optimal conditions using tri-*n*-propylamine (*n*-Pr_3_N) as the base, no solvent, and 140 °C under aerobic conditions. In general, the reaction performed with aryl bromides and chlorides afforded the desired products, albeit with lower yield and requiring longer reaction times than those corresponding to the aryl iodides (Table 9, entry 1 vs. entries 8 and 11; entry 3 vs. entry 10; and entry 4 vs. 7) [71,122]. Interestingly, 3-bromopyridine was also converted into the desired product in a good yield (Table 9, entry 12). In general, the nature of substituents on aryl halides did not greatly influence the rate of the reaction or the yield of the product. The recyclability of the catalyst was demonstrated by performing 5 consecutive runs for the reaction with iodobenzene, which was completed in 5 min. The isolated yield of the desired product was maintained at approximately 90% for the first 3 runs and decreased gradually until 75% in the last run [61]. Part of this continuing decrease in the yield could be understood considering the partial loss of catalyst during the recovery process that involved filtration, washing, and drying.

Four years later, the same authors from the previous example published the study of the same reaction between aryl/hetero aryl halides with *n*-butyl acrylate and styrene but catalyzed by **LSA-Fe_3_O_4_NPs-PdNPs** (a core-shell nanocomposite, see Section 2.3) [80]. Under the same reaction conditions (i.e., *n*-Pr_3_N, 140 °C, no solvent, catalyst 0.05 g) and carefully comparing the results previously obtained with **LSA-PdNPs [61]**, the new catalyst allowed, in general, the desired products to be obtained with comparable yields. In some cases, especially with chloro and bromo derivatives, the yield could be increased by 5 and 10%, respectively, using significantly shorter reaction times.

#### 3.3.2. LSA-PdNPs: Carbonized Lignin-Based Catalyst

1. Suzuki–Miyaura Coupling Reaction

In 2009, Guillén and coworkers reported the use of **LACT-Pd** (i.e., palladium supported on mesoporous activated carbon obtained from lignin, see Section 2.3) as a catalyst for the Suzuki–Miyaura coupling between arylbromides and arylboronic acids under mild conditions [50]. In this short study, the reactions were carried out for 21 h using 4-bromotoluene (i.e., referring to the head scheme in Table 7: R^1^ = Me, X = Br) and three substituted phenylboronic acids (i.e., referring to the head scheme in Table 7: R^2^ = OMe, NO_2_, H) in the presence of K_2_CO_3_ in DMA/water (20:1) at 70 °C. All desired cross-coupling products were obtained in a 85–98% yield. The homocoupling product was observed in only a 5% yield in the case of 4-methoxyphenylboronic acid, probably due to the electron-donating effect of the OMe group. Furthermore, **LACT-Pd** could be recovered by simple filtration and reused without a significant loss of activity. It is also worth mentioning that the carbon catalyst used in that study had a palladium content as low as 0.5%, which is 10–40 times lower than other Pd-supported active charcoal [123,124]. Moreover, triphenylphosphine was not added as a ligand. Thus, the presence of phosphorus groups such as C–O–P, C–P–O, and especially C_3_P on the surface of **LACT-Pd** is likely responsible for its catalytic activity in this reaction.

## 4. Conclusions

The advances summarized in this review pinpoint lignin as a green material to catalyze the formation of C–C bonds, either through its acid form **LSA** or as a support for metal ions and metal nanoparticles that direct the catalytic activity. There is no doubt that its heterogeneous nature provides advantages over other homogeneous catalysts. The examples reported thus far describe the successful recovery of the catalyst after the reaction by simple filtration, washing, and drying, allowing its reuse in subsequent reactions. In general, the slight yield losses observed in many of the reactions are most likely due to minor losses of the material during the recovery process rather than to the catalyst. Furthermore, the facile preparation of these lignin-derived catalysts and their low costs offer additional advantages compared to other catalysts. A variety of C–C formation reactions, such as condensations, Michael additions, alkylation of indoles, Pd-mediated cross-coupling reactions, etc., have been successfully carried out in the presence of **LSA**-based catalysts. Despite the promising results obtained thus far, there is still plenty of room for additional and more challenging studies. In general, the future of this area lies in performing more detailed studies regarding the kinetics of the processes (i.e., determination of rate constants, turn over number (TON) and turn over frequency (TOF) values) with a series of experiments designed in such a way that the results could be properly compared with those derived from the best catalysts described to date for the same transformations. In addition, it would be highly desirable in the future to test the catalytic potential of these materials in reactions that are poorly catalyzed by other systems to assess the real value of the lignin-based catalyst. Finally, the demonstration of high-scale synthesis and the inclusion of chiral auxiliaries or ligands into the lignin-based catalytic system would stimulate the study of stereoselective reactions of industrial interest, enabling a major development in the field.

## Figures and Tables

**Table 1:** **LSA**-catalyzed one-pot MCR [31] ^1^.

Entry	R^1^	R^2^	R^3^	Yield (%) ^2^
**Synthesis of benzoxanthenes (I)**				
1	Ph	Me	-	90
2	4-Me-Ph	Me	-	88
3	4-MeO-Ph	Me	-	86
4	4-Cl-Ph	Me	-	92
5	4-NO_2_-Ph	Me	-	93
6	*n*-C_3_H_7_	Me	-	82
7	Ph	H	-	89
8	4-Me-Ph	H	-	87
**Synthesis of 1-amidoalkyl-2-napththols (II)**				
9	Ph	-	Me	89
10	4-Me-Ph	-	Me	87
11	4-MeO-Ph	-	Me	84
12	4-Cl-Ph	-	Me	90
13	4-NO_2_-Ph	-	Me	91
14	Ph	-	Ph	90
15	4-Me-Ph	-	Ph	86
16	4-NO_2_-Ph	-	Ph	92
**Hantzch reaction (III)**				
17	4-Me-Ph	-	-	90
**Strecker reaction (IV)**				
18	4-Me-Ph	-	-	92

^1^ Typical reaction conditions: **Reaction I**: aldehydes (1.0 mmol), 2-naphthol (1.0 mmol), cyclic 1,3-dicarbonyl compounds (1.2 mmol), **LSA** (20 mg, containing −SO_3_H 0.038 mmol), 90 °C, 2 h. **Reaction II**: aldehydes (1.0 mmol), 2-naphthol (1.0 mmol), amides (1.2 mmol), **LSA** (20 mg, containing −SO_3_H 0.038 mmol), 90 °C, 2 h. **Reaction III**: 4-methylbenzaldehyde (1.0 mmol), ethylacetoacetate (2.0 mmol), NH_4_OAc (1.5 mmol), **LSA** (20 mg, containing −SO_3_H 0.038 mmol), 90 °C, 2 h. **Reaction IV**: 4-methylbenzaldehyde (1.0 mmol), aniline (1.5 mmol), TMSCN (1.2 mmol), **LSA** (15 mg, containing −SO_3_H 0.028 mmol), 90 °C, 1 h. ^2^ Isolated yield.

**Table 2:** **LS-Sc^3+^** promoted the Michael addition/dehydration tandem reaction between 3-acetyl-2-hydroxy-2-methylchromenes and indoles [33] ^1^.

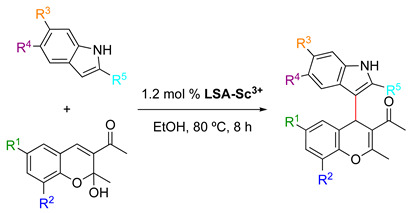							
Entry	Catalyst	R^1^	R^2^	R^3^	R^4^	R^5^	Yield (%) ^2^
1	**LSA-Sc^3+^**	Br	H	H	H	H	93 (90)
2	**LSA-Sc^3+^**	Br	H	H	H	Me	98
3	**LSA-Sc^3+^**	Br	H	H	OMe	H	97
4	**LSA-Sc^3+^**	Br	H	H	Br	H	61
5	**LSA-Sc^3+^**	Br	H	Me	H	H	93
6	**LSA-Sc^3+^**	Br	H	H	H	Ph	85 ^3^
7	**LSA-Sc^3+^**	Cl	H	H	H	H	96
8	**LSA-Sc^3+^**	Cl	H	H	H	Me	96
9	**LSA-Sc^3+^**	H	H	H	H	H	93
10	**LSA-Sc^3+^**	OMe	H	H	H	H	86
11	**LSA-Sc^3+^**	*t*Bu	H	H	H	H	96
12	**LSA-Sc^3+^**	H	Me	H	H	H	68
13	**LSA-Sc^3+^**	H	OMe	H	H	H	61
14	**LSA-Sc^3+^**	Me	Me	H	H	H	83
15	**SiO_2_-Sc^3+^**	Br	H	H	H	H	92 (50)
16	**resin-Sc^3+^**	Br	H	H	H	H	87 (63)

^1^ Reaction conditions: 3-acetyl-2-hydroxy-2-methylchromene derivative (0.5 mmol), indole derivative (0.6 mmol), **LSA-Sc^3+^** (1.2 mol%), EtOH (1 mL), 80 °C, 8 h. Isolated yields. The yield in parentheses shows the reuse of the catalyst in the third run. ^2^ Isolated yield. ^3^ Catalyst amount = 2 mol%. 1-Methyl-2-phenyl-1*H*-indole was used as the Michael donor.

**Table 3:** **LSA-Sc^3+^** catalyzed electrophilic ring-opening reactions of 2-butoxy-3,4-dihydropyrans with indoles [33] ^1^.

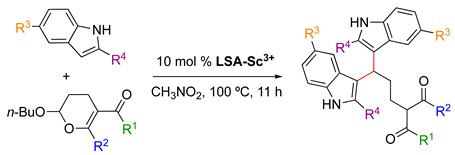					
Entry	R^1^	R^2^	R^3^	R^4^	Yield (%) ^2^
1	OEt	Me	H	H	89 (88)
2	OEt	Me	Br	H	86
3	OEt	Ph	H	H	89
4	OEt	Ph	Br	H	63
5	OMe	Me	H	H	87
6	OMe	Me	H	Me	80
7	OMe	Me	Br	H	90
8	Me	Me	H	H	95
9	Me	Me	Br	H	96
10	OCH_2_CH_2_OMe	Me	H	H	87

^1^ Reaction conditions: 2-butoxy-3,4-dihydropyran derivative (0.5 mmol), indole derivative (1.25 mmol), **LSA-Sc^3+^** (10 mol%), CH_3_CN_2_ (1 mL), 100 °C, 11 h. Isolated yields. The yield in parentheses shows reuse of the catalyst in the third run. ^2^ Isolated yield.

**Table 4:** **LSA-FAS-Cu^2+^** catalyzed three-component reactions of 4-aminoindoles, alkynes, and aldehydes [34] ^1^.

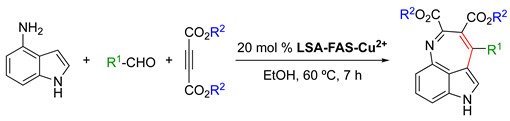			
Entry	R^1^	R^2^	Yield (%) ^2^
1	4-Me-Ph	Et	86
2	Ph	Et	61
3	4-Br-Ph	Et	46
4	4-CF_3_-Ph	Et	64
5	2-OMe-Ph	Et	55
6	2-NaPh	Et	60
7	2-Cl,3-Cl-Ph	Et	42
8	(3-OMe,4-OMe,5-OMe)-Ph	Et	37
9	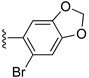	Et	64
10	CH_2_CH_2_-Ph	Et	45
11	Me	Et	80
12	Pr	Et	74
13	CH_2_*t*Bu	Et	71
14	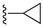	Et	77
15	4-Me-Ph	Me	65

^1^ Reaction conditions: 4-aminoindole/aldehyde/alkyne = 1.5:1:1.5, **LSA-FAS-Cu^2+^** (20 mol%), EtOH (1 mL), 60 °C, 7 h. ^2^ Isolated yield.

**Table 5:** **LSA-FAS-Cu^2+^** catalyzed the reaction of acetophenones and 1,3-diaminopropane, affording 2-arylpyridine derivatives [34] ^1^.

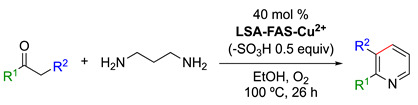			
Entry	R^1^	R^2^	Yield (%) ^2^
1	Ph	H	74
2	4-Me-Ph	H	71
3	4-*n*-Pr-Ph	H	70
4	4-*n*-amyl-Ph	H	68
5	4-*t*-Bu-Ph	H	63
6	4-Cy-Ph	H	60
7	4-SMe-Ph	H	82
8	4-OMe-Ph	H	65
9	4-OBn-Ph	H	67
10	4-Ph-Ph	H	63
11	4-F-Ph	H	45
12	4-Cl-Ph	H	40
13	4-I-Ph	H	39
14	3-Me-Ph	H	65
15	2-NaPh	H	58
16	(3-Me,5-Me)-Ph	H	68
17	(3-OMe,4-OMe)-Ph	H	56
18	(3-OMe,4-OMe,5-OMe)-Ph	H	73
19	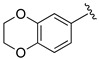	H	64
20	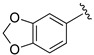	H	71
21	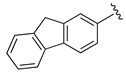	H	69
22	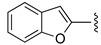	H	76
23	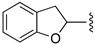	H	75

^1^ Reaction conditions: ketone (0.2 mmol), 1-3-diaminopropane (0.6 mmol), acidified **LSA-FAS-Cu^2+^** [(40 mol%), −SO_3_H (0.5 equiv)], O_2_ (1 atm), EtOH (1 mL), 100 °C, 26 h. Isolated yields. Note: For the sake of clarity, the group R^2^ is indicated in the reaction scheme, albeit R^2^ = H for all the examples. ^2^ Isolated yield.

**Table 6:** **LSA-FAS-Cu^2+^**-catalyzed synthesis of aminonaphthalene derivatives [34] ^1^.

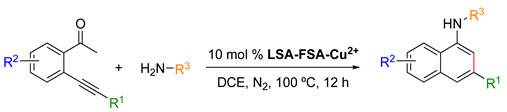				
Entry	R^1^	R^2^	R^3^	Yield (%) ^2^
1	Ph	H	4-Me-Ph	81
2	Ph	H	4-OMe-Ph	85
3	Ph	H	4-*t*-Bu-Ph	79
4	Ph	H	4-Br-Ph	55
5	Ph	H	(3-OMe,4-OMe)-Ph	66
6	Ph	H	1-naphthalene	70
7	Ph	H	Bn	61
8	Ph	H	*n*-Bu	83
9	Ph	H	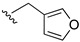	88
10	Ph	H	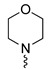	81
11	Ph	Cl	4-Me-Ph	78
12	Ph	F	4-Me-Ph	63
13	4-F-Ph	H	4-Me-Ph	71
14	*n*-hexyl	H	4-Me-Ph	54
15	cyclohexenyl	H	4-MePh	42
16	Ph	H	4-NO_2_Ph	n.d.
17	4-OMe-Ph	H	4-Me-Ph	n.d.

^1^ Reaction conditions: ketone (0.2 mmol), amine (0.24 mmol), **LSA-FAS-Cu^2+^** (10 mol%), DCE (1 mL), 100 °C, 12 h. ^2^ Isolated yield.

**Table 7:** **LSA-PdNPs** catalyzed the Suzuki–Miyaura cross-coupling reaction between aryl halides and phenyl boronic acids [71] ^1^.

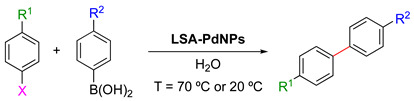							
Entry	X	R^1^	R^2^	Time (h)	T (°C)	Aryl Halide Conv. (%) ^2^	Yield (%) ^2^
1	I	H	H	5	70	100	100
2	I	H	H	12	20	100	100
3	CH=CH_2_	H	H	0.5	70	100	100
4	CH=CH_2_	H	H	2	20	100	100
5	I	NH_2_	H	12	70	70	28
6	I	NH_2_	H	12	20	0	-
7	Br	H	H	12	70	100	80
8	Br	H	H	12	20	15	10
9	Br	OMe	H	12	70	90	20
10	Br	OMe	H	12	20	90	25
11	I	H	CH_2_OH	12	70	100	100
12	I	H	CH_2_OH	12	20	84	84
13	Br	H	CH_2_OH	12	70	95	75
14	Br	H	CH_2_OH	12	20	20	15
15	Cl	H	CH_2_OH	12	70	10	5
16	Cl	H	CH_2_OH	12	20	18	12

^1^ Reaction conditions: aryl halide (0.5 mmol), phenyl boronic acid derivative (0.5 mmol), K_2_CO_3_ (1.75 mmol), H_2_O (5 mL), aryl halide: **LSA-PdNPs** = 450:1 (molar ratio). Reaction times and temperatures as indicated. ^2^ Calculated by GC-MS.

**Table 8:** **LSA-PdNPs** catalyzed the Heck reaction between aryl halides and styrene in aqueous alkaline conditions [71].

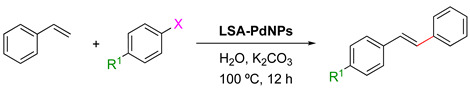			
Entry	X	R^1^	Yield (%) ^2^
1	I	H	100
2	I	NH_2_	50
3	I	CO_2_H	80 ^3^
4	Br	H	30 ^4^
5	Br	OMe	50

^1^ Reaction conditions: aryl halide (0.5 mmol), alkene (0.5 mmol), K_2_CO_3_ (1.75 mmol), H_2_O (5 mL), aryl halide: **LSA-PdNPs** = 450:1 (molar ratio), 100 °C, 12 h. ^2^ Calculated by GC-MS. ^3^ Biphenyl was also found. ^4^ Biphenyl was also found.

**Table 9:** **LSA-PdNPs** catalyzed the Heck reaction between *n*-butyl acrylate and aryl halides [61] ^1^.

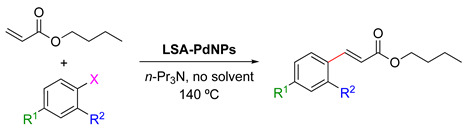					
Entry	X	R^1^	R^2^	Reaction Time	Yield (%) ^2^
1	I	H	H	5 min	94
2	I	H	Me	30 min	91
3	I	Me	H	15 min	92
4	I	OMe	H	20 min	90
5	I	NO_2_	Me	25 min	91
6	Br	NO_2_	H	1.2 h	90
7	Br	OMe	H	18 h	56
8	Br	H	H	17 h	75
9	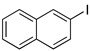	-	H	30 min	90
10	Br	Me	H	17 h	55
11	Cl	H	H	18 h	70
12	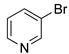	H	H	6 h	60

^1^ Reaction conditions: aryl halide (1 mmol), *n*-butyl acrylate (1.5 mmol), *n*-Pr_3_N (1.5 mmol), **LSA-PdNPs** (0.05 g; 0.0028 mmol of Pd). ^2^ Isolated yields.

## Data Availability

The data generated from the study are presented and discussed in the manuscript.

## References

[1] Bhunia A., Yetra S.R., Biju A.T. (2012). Recent advances in transition-metal-free carbon–carbon and carbon–heteroatom bond-forming reactions using arynes. Chem. Soc. Rev..

[2] Brahmachari G. (2016). Design of organic transformations at ambient conditions: Our sincere efforts to the cause of green chemistry practice. Chem. Rec..

[3] Cernansky R. (2015). Chemistry: Green refill. Nature.

[4] Farrán A., Cai C., Sandoval M., Xu Y., Liu J., Hernáiz M.J., Linhardt R.J. (2015). Green solvents in carbohydrate chemistry: From raw materials to fine chemicals. Chem. Rev..

[5] Klemm D., Heublein B., Fink H.-P., Bohn A. (2005). Cellulose: Fascinating biopolymer and sustainable raw material. Angew. Chem. Int. Ed..

[6] Wei W.-L., Zhu H.-Y., Zhao C.-L., Huang M.-Y., Jiang Y.-Y. (2004). Asymmetric hydrogenation of furfuryl alcohol catalyzed by a biopolymer–metal complex, silica-supported alginic acid–amino acid–Pt complex. React. Funct. Polym..

[7] Guibal E. (2005). Heterogeneous catalysis on chitosan-based materials: A review. Prog. Polym. Sci..

[8] Huang K., Xue L., Hu Y.C., Huang M.Y., Jiang Y.Y. (2002). Catalytic behaviors of silica-supported starch–polysulfosiloxane–Pt complexes in asymmetric hydrogenation of 4-methyl-2-pentanone. React. Funct. Polym..

[9] Gopiraman M., Fujimori K., Kim B., Kim I. (2013). Structural and mechanical properties of cellulose acetate/graphene hybrid nanofibers: Spectroscopic investigations. Express Polym. Lett..

[10] Ahvazi B., Wojciechowicz O., Ton-That T.-M., Hawari J. (2011). Preparation of lignopolyols from wheat straw soda lignin. J. Agric. Food Chem..

[11] Agrawal A., Kaushik N., Biswas S. (2014). Derivatives and applications of lignin–An insight. Sci. Tech. J..

[12] Luo H., Abu-Omar M.M. (2017). Chemicals from lignin. Encyclopedia of Sustainable Technologies.

[13] Liu W.-J., Jiang H., Yu H.-Q. (2015). Thermochemical conversion of lignin to functional materials: A review and future directions. Green Chem..

[14] Norgren M., Edlund H. (2014). Lignin: Recent advances and emerging applications. Curr. Opin. Colloid Interface Sci..

[15] Thakur V.K., Thakur M.K., Raghavan P., Kessler M.R. (2014). Progress in green polymer composites from lignin for multifunctional applications: A review. ACS Sustain. Chem. Eng..

[16] Ten E., Vermerris W. (2015). Recent developments in polymers derived from industrial lignin. J. Appl. Polym. Sci..

[17] Thakur V.K., Thakur M.K. (2015). Recent advances in green hydrogels from lignin: A review. Int. J. Biol. Macromol..

[18] Kuhad R.C., Singh A. (2007). Lignocellulose Biotechnology: Future Prospects.

[19] Lu Y., Sun Q.F., Yang D.J., She X.L., Yao X.D., Zhu G.S., Liu Y.X., Zhao H.J., Li J. (2012). Fabrication of mesoporous lignocellulose aerogels from wood via cyclic liquid nitrogen freezing–thawing in ionic liquid solution. Mater. Chem..

[20] Dorrestijn E., Laarhoven L.J.J., Arends I., Mulder P. (2000). The occurrence and reactivity of phenoxyl linkages in lignin and low rank coal. J. Anal. Appl. Pyrolysis.

[21] Rinaldi R., Jastrzebski R., Clough M.T., Ralph J., Kennema M., Bruijnincx P.C.A., Weckhuysen B.M. (2016). Paving the way for lignin: Recent advances in bioengineering, biorefining and catalysis. Angew. Chem. Int. Ed..

[22] Fengel D., Wegener G. (2003). Wood: Chemistry, Ultrastructure, Reactions.

[23] Crestini C., Lange H., Sette M., Argyropoulos D.S. (2017). On the structure of softwood Kraft lignin. Green Chem..

[24] Vishtal A., Kraslawski A. (2011). Challenges in industrial applications of technical lignins. Bioresources.

[25] Zhu Y., Li Z., Chen J. (2019). Applications of lignin-derived catalysts for green synthesis. Green Energy Environ..

[26] Li C., Zhao X., Wang A., Huber G.W., Zhang T. (2015). Catalytic transformation of lignin for the production of chemicals and fuels. Chem. Rev..

[27] Mennani M., Kasbaji M., Benhamou A.A., Boussetta A., Mekkaoui A.A., Grimig N., Moubarik A. (2023). Current approaches, emerging developments and functional prospects for lignin-based catalysts—A review. Green Chem..

[28] Aro T., Fatehi P. (2017). Production and application of lignosulfonates and sulfonated lignin. ChemSusChem.

[29] Gao W., Inwood J.P., Fatehi P. (2019). Sulfonation of phenolated kraft lignin to produce water soluble products. J. Wood Chem. Technol..

[30] Li J., Li H., Yuan Z., Fang J., Chang L., Zhang H., Li C. (2019). Role of sulfonation in lignin-based material for adsorption removal of cationic dyes. Int. J. Biol. Macromol..

[31] Chen W., Peng X.W., Zhong L.X., Li Y., Sun R.C. (2015). Lignosulfonic acid: A renewable and effective biomass-based catalyst for multicomponent reactions. ACS Sustain. Chem. Eng..

[32] Yamaguchi D., Kitano M., Suganuma S., Nakajima K., Kato H., Hara M. (2009). Hydrolysis of cellulose by a solid acid catalyst under optimal reaction conditions. J. Phys. Chem. C.

[33] Sun S., Bai R., Gu Y. (2014). From waste biomass to solid support: Lignosulfonate as a cost-effective and renewable supporting material for catalysis. Chem. Eur. J..

[34] Lai B., Ye M., Liu P., Li M., Bai R., Gu Y. (2020). A novel and robust heterogeneous Cu catalyst using modified lignosulfonate as support for the synthesis of nitrogen-containing heterocycles. Beilstein J. Org. Chem..

[35] Maggi R., Shiju N.R., Santacroce V., Maestri G., Bigi F., Rothenberg G. (2016). Silica-supported sulfonic acids as recyclable catalyst for esterification of levulinic acid with stoichiometric amounts of alcohols. Beilstein J. Org. Chem..

[36] Gao S., Luo T., Zhou Q., Luo W., Li H., Jing L. (2018). Surface sodium lignosulphonate-immobilized sawdust particle as an efficient adsorbent for capturing Hg^2+^ from aqueous solution. J. Colloid Interface Sci..

[37] Jakab E., Faix O., Till F., Székely T. (1993). The effect of cations on the thermal decomposition of lignins. J. Anal. Appl. Pyrolysis.

[38] Fiorani G., Perosa A., Selva M. (2018). Dimethyl carbonate: A versatile reagent for a sustainable valorization of renewables. Green Chem..

[39] Li S., Liu S., Fu Z., Li Q., Wu C., Guo W. (2017). Surface modification and characterization of carbon black by sodium lignosulphonate. Surf. Interface Anal..

[40] Li H., Cheng R., Liu Z., Du C. (2019). Waste control by waste: Fenton–like oxidation of phenol over Cu modified ZSM–5 from coal gangue. Sci. Total Environ..

[41] Dong X., Ren B., Sun Z., Li C., Zhang X., Kong M., Zheng S., Dionysiou D.D. (2019). Monodispersed CuFe_2_O_4_ nanoparticles anchored on natural kaolinite as highly efficient peroxymonosulfate catalyst for bisphenol A degradation. Appl. Catal. B..

[42] Ghodsinia S.S.E., Akhlaghinia B. (2019). CuI anchored onto mesoporous SBA-16 functionalized by aminated 3-glycidyloxypropyltrimethoxysilane with thiosemicarbazide (SBA-16/GPTMS-TSC-Cu I): A heterogeneous mesostructured catalyst for S-arylation reaction under solvent-free conditions. Green Chem..

[43] Zhao J., Wang J., Brock A.J., Zhu H. (2022). Plasmonic heterogeneous catalysis for organic transformations. J. Photochem. Photobiol..

[44] Gellé A., Jin T., de la Garza L., Price G.D., Besteiro L.V., Moores A. (2019). Applications of plasmon-enhanced nanocatalysis to organic transformations. Chem. Rev..

[45] Ndolomingo M.J., Bingwa N., Meijboom R. (2020). Review of supported metal nanoparticles: Synthesis methodologies, advantages and application as catalysts. J. Mater. Sci..

[46] Landge V.K., Sonawane S.H., Manickam S., Babu G.U.B., Boczkaj G. (2021). Ultrasound-assisted wet-impregnation of Ag–Co nanoparticles on cellulose nanofibers: Enhanced catalytic hydrogenation of 4-nitrophenol. J. Environ. Chem. Eng..

[47] Khan S.A., Ismail M., Anwar Y., Farooq A., Al Johny B.O., Akhtar K., Sha Z.A., Nadeen M., Raza M.A., Asiri A.M. (2019). A highly efficient and multifunctional biomass supporting Ag, Ni, and Cu nanoparticles through wetness impregnation for environmental remediation. Green Process. Synth..

[48] Rodrigues T.S., Zhao M., Yang T.H., Gilroy K.D., da Silva A.G., Camargo P.H., Xia Y. (2018). Synthesis of colloidal metal nanocrystals: A comprehensive review on the reductants. Eur. J. Chem..

[49] Bonardd S., Saldías C., Ramírez O., Radić D., Recio F.J., Urzúa M., Leiva A. (2019). A novel environmentally friendly method in solid phase for in situ synthesis of chitosan-gold bionanocomposites with catalytic applications. Carbohydr. Polym..

[50] Guillen E., Rico R., López-Romero J.M., Bedia J., Rosas J.M., Rodríguez-Mirasol J., Cordero T. (2009). Pd-activated carbon catalysts for hydrogenation and Suzuki reactions. Appl. Catal..

[51] Kaneko K., Ishii C. (1992). Superhigh surface area determination of microporous solids. Colloids Surf..

[52] Stoeckli F., López-Ramón M.V., Hugi-Cleary D., Guillot A. (2001). Micropore sizes in activated carbons determined from the Dubinin–Radushkevich equation. Carbon.

[53] Martin-Gullon I., Marco-Lozar J.P., Cazorla-Amorós D., Linares-Solano A. (2004). Analysis of the microporosity shrinkage upon thermal post-treatment of H3PO4 activated carbons. Carbon.

[54] Cordero T., Rodriguez-Mirasol J., Tancredi N., Piriz J., Vivo G., Rodriguez J.J. (2002). Influence of surface composition and pore structure on Cr (III) adsorption onto activated carbons. Ind. Eng. Chem. Res..

[55] Rosas J.M., Bedia J., Rodríguez-Mirasol J., Cordero T. (2008). Preparation of hemp-derived activated carbon monoliths. Adsorption of water vapor. Ind. Eng. Chem. Res..

[56] Puziy A.M., Poddubnaya O.I., Martínez-Alonso A., Suárez-García F., Tascón J.M. (2005). Surface chemistry of phosphorus-containing carbons of lignocellulosic origin. Carbon.

[57] Puziy A.M., Poddubnaya O.I., Ziatdinov A.M. (2006). On the chemical structure of phosphorus compounds in phosphoric acid-activated carbon. Appl. Surf. Sci..

[58] Wu X., Radovic L.R. (2006). Inhibition of catalytic oxidation of carbon/carbon composites by phosphorus. Carbon.

[59] Bedia J., Rosas J.M., Marquez J., Rodriguez-Mirasol J., Cordero T. (2009). Preparation and characterization of carbon based acid catalysts for the dehydration of 2-propanol. Carbon.

[60] Rosas J.M., Bedia J.R.M.J., Rodríguez-Mirasol J., Cordero T. (2009). HEMP-derived activated carbon fibers by chemical activation with phosphoric acid. Fuel.

[61] Marulasiddeshwara M.B., Kumar P.R. (2016). Synthesis of Pd (0) nanocatalyst using lignin in water for the Mizoroki–Heck reaction under solvent-free conditions. Int. J. Biol. Macromol..

[62] Moulder J.F., Stickle W.F., Sobol P.E., Bomben K.D., Chastain J., King R.C. (1995). Handbook of X-ray Photoelectron Spectroscopy.

[63] De Pedro Z.M., Gómez-Sainero L.M., González-Serrano E., Rodríguez J.J. (2006). Gas-phase hydrodechlorination of dichloromethane at low concentrations with palladium/carbon catalysts. Ind. Eng. Chem. Res..

[64] Radkevich V.Z., Senko T.L., Wilson K., Grishenko L.M., Zaderko A.N., Diyuk V.Y. (2008). The influence of surface functionalization of activated carbon on palladium dispersion and catalytic activity in hydrogen oxidation. Appl. Catal. A.

[65] Dury F., Gaigneaux E.M., Ruiz P. (2003). The active role of CO_2_ at low temperature in oxidation processes: The case of the oxidative dehydrogenation of propane on NiMoO_4_ catalysts. Appl. Catal. A.

[66] Demoulin O., Navez M., Mugabo J.L., Ruiz P. (2007). The oxidizing role of CO_2_ at mild temperature on ceria-based catalysts. Appl. Catal. B Environ..

[67] Calvo L., Mohedano A.F., Casas J.A., Gilarranz M.A., Rodríguez J.J. (2004). Treatment of chlorophenols-bearing wastewaters through hydrodechlorination using Pd/activated carbon catalysts. Carbon.

[68] Datye A.K., Xu Q., Kharas K.C., McCarty J.M. (2006). Particle size distributions in heterogeneous catalysts: What do they tell us about the sintering mechanism?. Catal. Today.

[69] McKee D.W., Thrower P.A. (1991). Oxidation Protection of Carbon Materials.

[70] Labruquère S., Pailler P., Naslain R., Desbat B. (1998). Oxidation inhibition of carbon fibre preforms and C/C composites by H_3_PO_4_. J. Eur. Ceram. Soc..

[71] Coccia F., Tonucci L., d’Alessandro N., D’Ambrosio P., Bressan M. (2013). Palladium nanoparticles, stabilized by lignin, as catalyst for cross-coupling reactions in water. Inorg. Chim. Acta.

[72] Teranishi T., Miyake M. (1998). Size control of palladium nanoparticles and their crystal structures. Chem. Mater..

[73] Navaladian S., Viswanathan B., Varadarajan T.K., Viswanath R.P. (2009). A rapid synthesis of oriented palladium nanoparticles by UV irradiation. Nanoscale Res. Lett..

[74] Wang B., Yang G., Chen J., Fang G. (2020). Green synthesis and characterization of gold nanoparticles using lignin nanoparticles. Nanomaterials.

[75] Khazaei A., Rahmati S., Hekmatian Z., Saeednia S. (2013). A green approach for the synthesis of palladium nanoparticles supported on pectin: Application as a catalyst for solvent-free Mizoroki–Heck reaction. J. Mol. Catal. A Chem..

[76] Li Y., Hong X.M., Collard D.M., El-Sayed M.A. (2000). Suzuki cross-coupling reactions catalyzed by palladium nanoparticles in aqueous solution. Org. Lett..

[77] Szilvia P., Rita P., Imre D. (2007). Formation and stabilization of noble metal nanoparticles. Croat. Chem. Acta.

[78] Roy P.S., Bagchi J., Bhattacharya S.K. (2010). Synthesis of polymer-protected palladium nanoparticles of contrasting electrocatalytic activity: A comparative study with respect to reflux time and reducing agents. Colloids Surf. A Physicochem. Eng. Asp..

[79] Zhu W., Yang H., Yu Y., Hua L., Li H., Feng B., Hou Z. (2011). Amphiphilic ionic liquid stabilizing palladium nanoparticles for highly efficient catalytic hydrogenation. PCCP.

[80] Madrahalli Bharamanagowda M., Panchangam R.K. (2020). Fe_3_O_4_-Lignin@ Pd-NPs: A highly efficient, magnetically recoverable and recyclable catalyst for Mizoroki-Heck reaction under solvent-free conditions. Appl. Organomet. Chem..

[81] Zamani F., Hosseini S.M. (2014). Palladium nanoparticles supported on Fe_3_O_4_/amino acid nanocomposite: Highly active magnetic catalyst for solvent-free aerobic oxidation of alcohols. Catal. Commun..

[82] Elazab H.A., Siamaki A.R., Moussa S., Gupton B.F., El-Shall M.S. (2015). Highly efficient and magnetically recyclable graphene-supported Pd/Fe_3_O_4_ nanoparticle catalysts for Suzuki and Heck cross-coupling reactions. Appl. Catal. A Gen..

[83] Banazadeh A., Pirisedigh A., Aryanasab F., Salimi H., Shafiei-Haghighi S. (2015). Novel synthesis and characterization of Fe_3_O_4_@ silica–palladium nanocatalyst: A highly active and reusable heterogeneous catalyst for Heck cross-coupling reactions. Inorg. Chim. Acta.

[84] Vaddula B.R., Saha A., Leazer J., Varma R.S. (2012). A simple and facile Heck-type arylation of alkenes with diaryliodonium salts using magnetically recoverable Pd-catalyst. Green Chem..

[85] Gu Y. (2012). Multicomponent reactions in unconventional solvents: State of the art. Green Chem..

[86] John S.E., Gulati S., Shankaraiah N. (2021). Recent advances in multi-component reactions and their mechanistic insights: A triennium review. Org. Chem. Front..

[87] Li M., Taheri A., Liu M., Sun S., Gu Y. (2014). Three-component reactions of aromatic aldehydes and two different nucleophiles and their leaving ability-determined downstream conversions of the products. Adv. Synth. Catal..

[88] Wang H., Lu L., Zhu S., Li Y., Cai W. (2006). The phototoxicity of xanthene derivatives against *Escherichia coli*, *Staphylococcus aureus*, and *Saccharomyces cerevisiae*. Curr. Microbiol..

[89] Seebach D., Matthews J.L. (1997). β-Peptides: A surprise at every turn. Chem. Commun..

[90] Wang Y.F., Izawa T., Kobayashi S., Ohno M. (1982). Stereo-controlled synthesis of (+)-negamycin from an acyclic homoallylamine by 1,3-asymmetric induction. J. Am. Chem. Soc..

[91] Zolfigol M.A., Khazaei A., Moosavi-Zare A.R., Zare A., Khakyzadeh V. (2011). Rapid synthesis of 1-amidoalkyl-2-naphthols over sulfonic acid functionalized imidazolium salts. Appl. Catal. A Gen..

[92] Zhang Q., Luo J., Wei Y. (2010). A silica gel supported dual acidic ionic liquid: An efficient and recyclable heterogeneous catalyst for the one-pot synthesis of amidoalkyl naphthols. Green Chem..

[93] Vo D., Matowe W.C., Ramesh M., Iqbal N., Wolowyk M.W., Howlett S.E., Knaus E.E. (1995). Syntheses, calcium channel agonistantagonist modulation activities, and voltage-clamp studies of isopropyl 1,4-dihydro-2,6-dimethyl-3-nitro-4-pyridinylpyridine-5-carboxylate racemates and enantiomers. J. Med. Chem..

[94] Kawase M., Shah A., Gaveriya H., Motohashi N., Sakagami H., Varga A., Molnár J. (2002). 3,5-Dibenzoyl-1,4-dihydropyridines: Synthesis and MDR reversal in tumor cells. Bioorg. Med. Chem..

[95] Chen Q., Huang W., Chen P., Peng C., Xie H., Zhao Z.K., Sohail M., Bao M. (2015). Synthesis of lignin-derived bisphenol catalyzed by lignosulfonic acid in water for polycarbonate synthesis. ChemCatChem.

[96] Bader A.R., Kontowicz A.D. (1954). γ,γ-Bis-(p-hydroxyphenyl)-valeric acid. J. Am. Chem. Soc..

[97] Bordwell F.G. (1988). Equilibrium acidities in dimethyl sulfoxide solution. Acc. Chem. Res..

[98] Das D., Lee J.-F., Cheng S. (2001). Sulfonic acid functionalized mesoporous MCM-41 silica as a convenient catalyst for bisphenol-A synthesis. Chem. Commun..

[99] Earle M.J., Seddon K.R. (2000). Ionic liquids. Grenn solvents for the future. Pure Appl. Chem..

[100] Kühbeck D., Saidulu G., Reddy K.R., Díaz D.D. (2011). Critical assessment of the efficiency of chitosan biohydrogel beads as recyclable and heterogeneous organocatalyst for C–C bond formation. Green Chem..

[101] Li M., Yang J., Gu Y. (2011). Manganese chloride as an efficient catalyst for selective transformations of indoles in the presence of a keto carbonyl group. Adv. Synth. Catal..

[102] Chen S., Ravichandiran P., El-Harairy A., Queneau Y., Li M., Gu Y. (2019). 4-Aminoindoles as 1,4-bisnucleophiles for diversity-oriented synthesis of tricyclic indoles bearing 3,4-fused seven-membered rings. Org. Biomol. Chem..

[103] Glaser C. (1869). Beiträge zur Kenntniss des Acetenylbenzols. Ber. Dtsch. Chem. Ges..

[104] Wang M.-Z., Zhou C.-Y., Wong M.-K., Che C.-M. (2010). Ruthenium-catalyzed alkylation of indoles with tertiary amines by oxidation of a sp3 C-H bond and lewis acid catalysis. Chem. Eur. J..

[105] Kothandapani J., Ganesan A., Ganesan S.S. (2015). Magnetically separable sulfonic acid catalysed one-pot synthesis of diverse indole derivatives. Tetrahedron Lett..

[106] Zheng S., Zhong Q., Mottamal M., Zhang Q., Zhang C., LeMelle E., McFerrin H., Wang G. (2014). Design, synthesis, and biological evaluation of novel pyridine-bridged analogues of combretastatin-A4 as anticancer agents. J. Med. Chem..

[107] Shintani R., Misawa N., Takano R., Nozaki K. (2017). Rhodium-catalyzed synthesis and optical properties of silicon-bridged arylpyridines. Chem. Eur. J..

[108] Kuzmina O.M., Steib K.A., Markiewicz J.T., Flubacher D., Knochel P. (2013). Ligand-accelerated iron- and cobalt-catalyzed cross-coupling reactions between N-heteroaryl halides and aryl magnesium reagents. Angew. Chem. Int. Ed..

[109] Donohoe T.J., Bower J.F., Basutto J.A., Fishlock L.P., Procopiou P.A., Callens C.K.A. (2009). Ring-closing metathesis for the synthesis of heteroaromatics: Evaluating routes to pyridines and pyridazines. Tetrahedron.

[110] Torborg C., Beller M. (2009). Recent Applications of Palladium-catalyzed coupling reactions in the pharmaceutical, agrochemical, and fine chemical industries. Adv. Synth. Catal..

[111] Miyaura N., Suzuki A. (1995). Palladium-catalyzed cross-coupling reactions of organoboron compounds. Chem. Rev..

[112] Sawoo S., Srimani D., Dutta P., Lahiri R., Sarkar A. (2009). Size controlled synthesis of Pd nanoparticles in water and their catalytic application in C–C coupling reactions. Tetrahedron.

[113] Kalbasi R.J., Mosaddegh N., Abbaspourrad A. (2012). A novel catalyst containing palladium nanoparticles supported on poly(2-hydroxyethyl methacrylate)/CMK-1: Synthesis, characterization and comparison with mesoporous silica nanocomposite. Appl. Catal. A.

[114] Martin R., Buchwald S.L. (2008). Palladium-catalyzed uzuki-Miyaura cross-coupling reactions employing dialkylbiaryl phosphine ligands. Acc. Chem. Res..

[115] Sonogashira K. (2002). Development of Pd–Cu catalyzed cross-coupling of terminal acetylenes with sp2-carbon halides. J. Organomet. Chem..

[116] Nicolaou K.C., Dai W.-M. (1991). Chemistry and Biology of the Enediyne Anticancer Antibiotics. Angew. Chem. Int. Ed. Engl..

[117] Keddie D.J., Fairfull-Smith K.E., Bottle S.E. (2008). The palladium-catalysed copper-free Sonogashira coupling of isoindoline nitroxides: A convenient route to robust profluorescent carbon–carbon frameworks. Org. Biomol. Chem..

[118] Sheffy F.K., Godschalx J.P., Stille J.K. (1984). Palladium-catalyzed cross coupling of allyl halides with organotin reagents: A method of joining highly functionalized partners regioselectively and stereospecifically. J. Am. Chem. Soc..

[119] Heck R.F. (1982). Palladium-catalyzed vinylation of organic halides. Org. React..

[120] Amatore C., Jutand A. (2000). Anionic Pd (0) and Pd (II) intermediates in palladium-catalyzed Heck and cross-coupling reactions. Acc. Chem. Res..

[121] Vries J.G. (2006). A unifying mechanism for all high-temperature Heck reactions. The role of palladium colloids and anionic species. J. Chem. Soc. Dalton Trans..

[122] Erdély M., Gogoll A. (2001). Rapid homogeneous-phase Sonogashira coupling reactions using controlled microwave heating. J. Org. Chem..

[123] Felpin F.X., Ayad T., Mitra S. (2006). Pd/C: An old catalyst for new applications—Its use for the Suzuki–Miyaura reaction. Eur. J. Org. Chem..

[124] Köhler K., Heidenreich R.G., Soomro S.S., Pröckla S.S. (2008). Supported palladium catalysts for Suzuki reactions: Structure-property relationships, optimized reaction protocol and control of palladium leaching. Adv. Synth. Catal..

